# Recovery of Polysaccharides from Red Grape Marc and White Grape Pomace by Degradation of Cell Walls by Enzymes with Different Activities

**DOI:** 10.3390/molecules30020213

**Published:** 2025-01-07

**Authors:** Ekhiñe Garaigordobil, Leticia Martínez-Lapuente, Zenaida Guadalupe, Silvia Pérez-Magariño, Belén Ayestarán

**Affiliations:** 1 Institute of Vine and Wine Sciences, ICVV (University of La Rioja, Government of La Rioja and CSIC), Finca La Grajera, 26007 Logroño, Spain; ekhine.garaigordobil@unirioja.es (E.G.); leticia.martinez@unirioja.es (L.M.-L.); zenaida.guadalupe@unirioja.es (Z.G.); 2 Agrarian Technological Institute of Castilla and León, Consejería de Agricultura y Ganadería, Ctra. Burgos Km 119, 47071 Valladolid, Spain; permagsi@itacyl.es

**Keywords:** grape polysaccharides from grapes, enzyme-assisted extraction, by-products, grape marc/pomace, molecular weight distribution of polysaccharides, purity, efficiency

## Abstract

The recovery of polysaccharides (PS) from red grape marc and white grape pomace by enzymatic degradation of their cell walls is an interesting green extraction technique that preserves the structure and bioactivity of PS. The type and dose of enzyme, and the liquid/solid (L/S) ratio in PS extraction were studied using four commercial enzymes. Four different doses per enzyme were used, with tartaric acid as solvent and L/S ratios of 1.3/1 and 4/1 for 24 h at 20 °C, compared with a control. The highest dose of enzyme E1, polygalacturonase + pectin lyase + pectin-methyl-esterase (with the highest activity) was the most effective in the degradation of high and medium molecular weight PS. At the lower L/S ratio, the fact that the highest dose of E1 degraded a higher percentage of high and medium molecular weight PS in the marc was explained by the difference in cell wall deconstruction between pomace and marc. The highest total PS purity was achieved in pomace with E1 at the maximum dose in both ratios, and in marc at the 1.3/1 ratio. The extraction efficiency of total PS was low for all enzymes. In the future, extraction with E1 combined with other green extraction techniques will be studied.

## 1. Introduction

The production of wine unfortunately generates massive amounts of agricultural waste. *Vitis vinifera* L. Viura and *Vitis vinifera* L. Tempranillo tinto are the most widely grown white (67.91%) and red (87.78%) grape varieties in the Qualified Designation of Origin (D.O.Ca) Rioja [1]. The primary waste product from wine and must production is grape marc/pomace, which represents approximately 20% to 30% of the total weight of the grapes used [2]. It is produced after alcoholic fermentation in the case of red grapes (marc), while for white grapes (pomace), it is produced before alcoholic fermentation. Grape marc/pomace is largely repurposed for composting; it can also be used for livestock feed, grape seed oil production, and it can be fermented and distilled to produce spirits. Nevertheless, current research continues to explore additional valorization strategies, as grape marc/pomace contains rich complexes of bioactive compounds, such as nitrogenous substances (serving as a substrate for fermentative catalysis and microorganism development), polyphenols (with antioxidant activity), and polysaccharides (including pectins, cellulose, hemicellulose, and xyloglucans) [3].

It is well known that polysaccharides from grape cell walls modify the technological and sensory properties of wine, just as commercial mannoproteins derived from yeast cell walls do. Currently, there is a growing focus on recovering polysaccharides from the cell walls of grape marc/pomace [4], due to the potential use of polysaccharide extracts as enological products to fine wine aroma and astringency. In the future, it is likely that grape polysaccharide extracts will replace exogenous additives used in must and wine, such as animal-derived proteins. Our group has demonstrated that the addition of polysaccharide extracts recovered from red grape marc or white grape pomace modulates the astringency of red wines with high phenolic content [5]. Furthermore, purified extracts rich in rhamnogalacturonans-II (RG-II), derived from both red wine and distillation by-products, are effective modulators of the aroma and astringency of white wines [6]. Likewise, it has been shown that extracts from pomace, red marc, white must, red must, white wine, and red wine modulate the aroma of white wine [7]. The extraction technique used in these studies (except for purified RG-II extract) was flash extraction [8]. However, it is necessary to study and optimize other extraction techniques for grape marc and grape pomace polysaccharides to obtain polysaccharide extracts with greater quantity and purity, which are essential for achieving an effect on wine quality.

Among the emerging green techniques for the extraction of bioactive compounds, Enzyme-Assisted Extraction (EAE) stands out. This technique improves process efficiency, enabling extractions at lower temperatures, which helps preserve the structure and bioactivity of polysaccharides [9,10]. Furthermore, EAE is an environmentally friendly technique, requiring simple procedures, lower investment costs, and reduced energy consumption [11].

The polysaccharides in grape marc/pomace originate from the pectocellulosic cell walls of grape berries [12]. These cell walls are divided into two layers: an outer layer of hemicellulose tightly bound to pectin and an inner layer rich in esterified pectin, which is more readily extractable [13]. Pectic polysaccharides include polysaccharides rich in arabinose and galactose (PRAG), which consist of arabinans, arabinogalactans (AG) and arabinogalactan proteins (AGP); rhamnogalacturonans type I and II (RG-I and RG-II); and homogalacturonans (HL) [8]. The strength of the association between pectin and the cellulose-xyloglucan network could be disrupted by treating the marc with cellulases and hemicellulases. Additionally, the degradation of the pectic fraction can be achieved using pectinases, especially endo-polygalacturonases, pectin methylesterases, and pectin lyases. Commercial maceration enzymes used in enology are complex mixtures of pectolytic enzymes (mainly containing polygalacturonase, pectin methyl esterase, and pectin lyase activities), cellulases, hemicelluloses, and acidic proteases [14,15]. One of the consequences of applying pectolytic enzymes in winemaking is the alteration in the composition and size of grape cell wall polysaccharides [16]. These authors found that the use of pectolytic enzymes enhanced the contribution of high molecular weight (≈200 kDa) mannose- and glucose-rich polysaccharides to the wine, removed intermediate-sized (≈40 kDa) arabinose-rich polysaccharides, and reduced the average molecular weight of rhamnogalacturonan II (6 kDa). The use of maceration enzymes to release compounds from grape solids is regulated by resolution OENO 13/04 and revision OENO 498-2013 [17]. In this regard, it is logical to employ commercial polysaccharide hydrolases under optimal experimental conditions to break down cell wall structures and recover grape polysaccharides.

Operational conditions such as reaction temperature, extraction time, system pH, enzyme concentration, substrate particle size, and liquid to solid (L/S) ratio are critical for the enzyme-assisted extraction process [18,19,20]. The objective of this work was to improve the extraction of polysaccharides from *Vitis vinifera* L. Tempranillo tinto grape marc and *Vitis vinifera* L. Viura grape pomace using cell wall-degrading enzymes. To optimize the enzymatic extraction procedure, this study analyses the influence of various parameters (type of commercial enzyme, enzyme dosage, and L/S ratio) on the composition and molecular size of the polysaccharides in the obtained extracts, as well as on the recovery of polysaccharides, expressed as extract purity and efficiency.

## 2. Results and Discussion

### 2.1. Quantification of Polysaccharides from Marc and Pomace Extracts

According to the activities of the commercial enzymes used in this study (Table 3, see Section 3.2) the breakdown of the pectic component of marc and pomace is performed by pectin lyase, which acts directly on the bonds between the galacturonic acid units of the pectic component of the skin cell wall. This enzyme catalyzes the β-elimination of de-esterified pectin, resulting in the formation of unsaturated 4,5-D-galacturonate as the final product through a transelimination process [21]. On the other hand, polygalacturonase catalyzes the hydrolytic cleavage of α-1,4-linkages of galacturonic acid, following the removal of methyl groups from pectin carried out by pectin methyl esterase [14]. Arabinase cleaves the bonds between arabinogalactan molecules, forming a branched chain of rhamnogalacturonan type I (RG-I). In addition, non-pectolytic enzymes also contribute to cell wall breakdown, with cellulase being responsible for degrading cellulose. Among these enzymes, glucanase releases glucose oligomers with β-1,4 linkages (cellulose) [22].

Table 1A shows that there was no linear relationship between the dose of E1 and the polysaccharide extraction. The highest E1 dose at both ratios was the most effective for extraction of TMS, TSP Pec and TSP. However, the extraction of HL, PRAG, RG-II, MAN and TSP Non Pec differed with the dose used at a 4/1 ratio. At this ratio, the highest extraction of HL was obtained with E1.1 and PRAG with E1.3, while E1.4 achieved the highest extraction of RG-II, MAN and TPS Non Pec. These results show that the extraction of the E1 enzyme depended on the dose but also on the ratio and type of polysaccharide. Only at the 4/1 ratio did the lowest dose of enzyme E3 (with higher number of activities, (Table 3, see Section 3.2) and E4.2 achieve higher values of TMS, PRAG, HL, TSP Pec, TSP Non Pec and TSP than the control, while the E3.1 at lower ratio (1.3/1) only extracted a higher content of HL than the control. Additionally, even the E4.1 resulted in higher HL values than the control at the 4/1 ratio. In these cases, the deconstruction effect on the polysaccharide network in the pomace cell wall depends not only on the dose, but also on the ratio. A higher ratio resulted in greater release of pectic and non-pectic families to the solvent. Several factors may explain this result. One possible reason may be due to the intensification of the driving force for the release of pectic and non-pectic polysaccharides from the pomace to the solution by increasing the expanded contact surface between solvent and pomace [23]. Another cause is probably that a high ratio promotes the swelling of the cell walls in the pomace and the loosening of cell wall structures, which would facilitate the solubilization of pectic and non-pectic polysaccharides [24].

The effect of increasing doses in E2 was not observed at either ratio, as no significant differences were found in the TMS, RG-II, MAN, PRAG, TSP Pec, TSP Non Pec, and TSP content compared to the control. The only exception was HL, where the lowest E2 dose at a 1.3/1 L/S ratio and dose 3 (0.08 μL of E/mL) at a 4/1 ratio achieved the highest HL values in the extracts.

In *Vitis vinifera* L. Viura pomace, the doses of E2 (polygalacturonase + pectin lyase), which acts by degrading pectin through glycosidic bond cleavage by pectin lyase and pectin depolymerization by polygalacturonase, were not sufficient to extract polysaccharides from the pomace in greater quantities than in the control, in any of the ratios used. These results do not agree with those of Osete-Alcaraz et al. [25], who observed that the polygalacturonase + pectin lyase enzyme was the most effective in depectinizing cell walls and increasing the exposure of grape hemicelluloses, thereby releasing the largest content of soluble polysaccharides. Probably, pomace cell walls retain an inner layer of pectin with a higher degree of methyl-esterification [26], which inhibits the action of polygalacturonase. As previously mentioned, the activity of polygalacturonase requires the prior removal of the methyl groups from pectin by pectin methyl-esterase [14] and the de-esterification of the pectin structure is necessary for pectin lyase to act [21]. However, Gao et al. [27] observed that the combination of endo-polygalacturonase and pectin methyl-esterase only unraveled the cell walls without depectinization. The results obtained for enzyme E1 confirmed that the highest activity of pectin methyl-esterase and enzyme doses (E1.4) are necessary for the extraction of pectic polysaccharides (TSP Pec). Under these conditions, the polysaccharide extracts obtained with E1 at both ratios showed the highest TSP Pec content, being from 44.49% to 51.19% (%*w*/*w* g of TSP Pec/100 g dried extract) (Table 1A), like the results obtained by Canalejo et al. [8] using the flash extraction technique. This fact indicates that E1 caused the highest depectinization of the pomace cell walls. However, it was also observed that the higher ratio (4/1) and dose E1.4 favored a high extraction of non-pectic polysaccharides (TSP Non Pec), similar to that obtained at the same ratio with the minimum dose of enzyme E3, and dose 2 (0.03 μL of E/mL) of E4. The greater depectinization of cell walls by the highest dose of polygalacturonase + pectin lyase + pectin methyl-esterase in E1 favored the exposure of cell wall hemicelluloses, releasing non-pectic polysaccharides, in similar quantity than that obtained with the minimum dose of E3 and dose 2 (0.03 μL of E/mL) of E4. Therefore, a higher solvent ratio was necessary to achieve a greater extraction of non-pectic polysaccharides from the pomace. The difference in activities between E1 and E3 (Table 3, see Section 3.2) was to break bonds between the arabinogalactan molecules of RG-I and release β-1,4 linked glucose oligomers (cellulose). The activities of E3 would explain why a lower dose of E3 than E4 (without arabinose activity) favored the release of non-pectic polysaccharides.

When comparing the same dose for the different enzymes, it was observed that the lowest dose of E1, with lower solvent consumption (ratio 1.3/1), achieved the highest release of TMS and TSP Pec (Table 1A). The higher solvent ratio (4/1) at both E1.1 and E3.1 extracted the largest amounts of TMS, PRAG, HL, TSP Pec, TSP Non Pec, and TSP, while the release of RG-II was significantly greater with E1.1 than with E3.1. The results for dose 2 (0.03 μL of E/mL) with the lower ratio (1.3/1) were not so clear. Under these conditions, the highest values of RG-II were obtained with E3.2, while E1.2 yielded the highest contents of HL and TSP Non Pec. With the higher ratio and dose 2, the highest contents of TMS, RG-II, TSP Pec, and TSP were obtained with E1, but the HL content was similar in E1.2 and E3.2; and E4.2 produced the highest extraction of MAN and TSP Non Pec. These results indicated that dose 2 of E1 needed a higher ratio to extract and solubilize the polysaccharides from the pomace to the solvent. No clear results were obtained for dose 3 (0.08 µL of E/mL) at the lower ratio, as there were no significant differences in the HL content among enzymes 1, 2, and 3, nor in the RG-II content among enzymes 1, 3, and 4, nor in the TSP content between enzymes 1 and 3. However, the results were clear with dose 3 at the higher ratio; the enzyme E1 achieved the highest release of TMS, RG-II, PRAG, TSP Pec, and TSP, while enzyme E3.3 was more effective in releasing MAN, HL, and TSP Non Pec. In general, with the maximum dose and at both ratios, enzyme E1 extracted higher contents of TMS, PRAG, TSP Pec, and TSP than the other enzymes. In general, these results indicated that, at equal doses, E1 at all doses and at the higher ratios was the most effective in the release of TMS, RG-II, PRAG, TSP Pec and TSP and that E1 also yielded the highest release of TSP Pec at both ratios.

The predominant polysaccharide families in pomace extracts were HL and PRAG, followed by RG-II and MAN. In general, the total content of pectic polysaccharides was higher than that of non-pectic polysaccharides (Table 1A).

In the EAE extracts of *Vitis vinifera* L. Tempranillo marc, the maximum extraction of TMS, TSP Pec, TSP Non Pec and TSP was achieved with the lowest dose of E2 and E3 enzymes at both ratios, while E4.1 only achieved a lower ratio (Table 1B). Comparing the enzymes E2, E3, and E4 at both ratios, the highest values of TMS, RG-II, PRAG, TSP Pec, TSP Non Pec, and TSP were obtained with the lowest dose of E2 at the lower ratio.

At the lower ratio (1.3/1), the highest E1 dose obtained higher contents of TMS, MAN, PRAG, HL, TSP Non Pec, and TSP than the control and than the lowest dose of the enzyme E2. The enzyme E1 at the higher ratio (4/1) exhibited different behavior in extraction. At 4/1 ratio, the highest content of TMS, RG-II, TSP Pec, and TSP were obtained at E1.2, while the highest concentrations of PRAG, HL, and TSP Non Pec were reached with the lowest dose (E1.1). It is interesting to note that the content of TMS, PRAG, TSP Pec, TSP Non Pec and TSP was higher with E1.4 at the 1.3/1 ratio than with E1.1 and E1.2 at the 4/1 ratio. The TSP content reached 78% (% *w*/*w*) with E1.4 at the lower ratio, a higher value than that reported by Canalejo et al. [8] using the flash extraction technique.

In summary, the ratio 1.3/1 was most favorable for the transfer of polysaccharides from the treated marc with the minimum doses of E2 and E4 to the solvent and with the maximum dose of E1. These results are contrary to those obtained by several authors [20,23,24,28]. These authors concluded that the pectin efficiency from different agricultural waste matrices increased as the liquid-to-solid ratio was increased, which favored mass transfer and reduces viscosity. However, higher L/S ratio also increases the difficulty of acidic ethanol precipitation of the polysaccharides [21]. This may explain the highest content of TSP in the marc observed with the lower L/S ratio and the minimum dose of E2 and E4, and the maximum dose of E1.

These results did not agree with the cell wall deconstruction caused by the dose effect of each enzyme obtained in *Vitis vinifera* L. Viura pomace. The literature indicates that red grape marc differs from white grape pomace in greater cell wall deconstruction, due to the crushing and fermentation–maceration characteristic of red winemaking, which leads to a higher extraction of polysaccharides from the skin compared to the pressing or crushing-pressing of white grapes [13,29]. Probably, the inner pectin layer of the marc cell wall has a lower degree of methyl-esterification, which explains the polygalacturonase + pectin lyase action of the minimum dose of E2, not observed in *Vitis vinifera* L. Viura pomace at any dose and at any ratio. As previously discussed, with the minimum dose of E2 (E2.1), the content of TMS, RG-II, MAN, PRAG, HL, TSP Pec, TSP Non Pec, and TSP was higher at ratio 1.3/1 than at ratio 4/1. It should be pointed out that even values of 12.47% (%*w*/*w*) of TSP Non Pec were extracted under the lowest dose of E2 and the lower ratio. This result indicated that the polygalacturonase + pectin lyase action also favored the exposure of cell wall hemicelluloses releasing non-pectic polysaccharides. Moreover, it is important to note that differences were observed in the effect of E1 at its maximum dose and lower ratio between pomace and marc. At these conditions, E1 was not able to release high contents of TSP Non Pec or MAN in the pomace. These results also confirmed that red grape marc differs from white grape pomace in a greater deconstruction of the cell wall.

At 4/1 ratio, the highest TMS, RG-II, PRAG, TSP Pec, TSP Non Pec, and TSP extraction was obtained with dose 2 of E1. The lower ratio was also more favorable for the transfer of TMS, RG-II, MAN, PRAG, HL, TSP Pec, TSP Non Pec, and TSP from the marc treated with the minimum doses of E2 and E4 to the solvent.

The lowest dose of E2 (E2.1) at the lower ratio and the lowest dose of E1 (E1.1) at the higher ratio were both most effective in releasing TMS, RG-II, MAN, PRAG, TSP Pec, TSP Non Pec, and TSP (Table 1B). These results did not agree with those observed in Viura pomace. E1.2 at both ratios extracted the highest amounts of TMS, RG-II, MAN, PRAG, HL, TSP Pec, TSP Non Pec, and TSP. E2.3 at the lower ratio was the most effective in the release of TMS, RG-II, MAN, PRAG, TSP Pec, TSP Non Pec, and TSP. However, E1 did not differ from E2 in the maximum extraction of TMS and TSP Pec. The maximum dose of E1 at both ratios achieved the highest content of TMS, RG-II, MAN, PRAG, HL, TSP Pec, TSP Non Pec and TSP.

In general, the majority polysaccharides in the extracts were PRAG and HL, followed by RG-II and MAN (Table 1B), and the pectic polysaccharide content was higher than non-pectic polysaccharides.

### 2.2. Distribution of the Molecular Weights of Polysaccharides by HPSEC-RID

The chromatograms obtained by HPSEC-RID on the Shodex column were divided into three fractions: high molecular weight fraction, HMW, (20–400 kDa), medium molecular weight fraction, MMW, (5.9–20 kDa), and low molecular weight fraction, LMW, (<5.9 kDa) (Figures S1 and S2). Table 2 shows the percentage of molecular weight distribution of polysaccharides from the EAE extracts of pomace and marc at the different doses of enzymes and in both ratios. The high and medium molecular weight fraction showed a lower percentage than the low molecular weight fraction. However, at both ratios, the percentage of low molecular weight polysaccharides was less than 58.91% in two pomace extracts and less than 35.10% in the marc in four extracts treated only by the enzyme E1 (Table 2). This result demonstrated that the action of polygalacturonase + pectinase + pectin-methylesterase of E1 was able to partially degrade high and medium molecular weight polysaccharide fragments to a greater extent and thus facilitate their extraction.

The high molecular weight peaks (Figures S1 and S2) corresponded to a complex mixture of arabinose- and galactose-rich polysaccharides (PRAG) and structural grape polysaccharides, such as celluloses and hemicelluloses [8]. PRAG exhibits a molar mass between 50 and 260 kg/mol in musts and wines [30] and represents the main family of grape-derived polysaccharides in wine. Cellulose microfibrils are composed of D-glucose linked by α-(1–4) bonds and are the primary component of cell wall polysaccharides [31]. Hemicellulosic polysaccharides consist primarily of xyloglucans with a main β-(1–4) D-glucan chain and side chains containing xylose, galactose, and fucose.

The medium molecular weight peak signals (Figures S1 and S2) would correspond to a complex mixture of dimers, mainly rhamnogalacturonan type II (RG-II), with an average molecular weight of 10–12 kg/mol in its dimeric form [32,33] and in its monomeric form (Mw ≈ 5 kg/mol) [17], together with fragments of PRAG and grape structural polysaccharides [8]. RG-II is present in the cell wall as either its monomeric or dimeric form, which is cross-linked by a borate diester, with the dimeric form predominating in wines [33]. RG-II consists of a short (1–4)-α-D-galacturonan backbone branched with four different chains containing mainly rhamnose, arabinose, and some rare carbohydrates such as 2-*O*-methyl-fucose, apiose, 2-*O*-methyl-xylose, Kdo (2-keto-3-deoxy-D-manno-octulosonic acid), DHA (3-deoxy-D-lyxo-heptulosaric acid), and aceric acid (3-C-carboxy-5-deoxy-L-xylose). RG-II content in skin tissue is three times higher than that in pulp tissue [34,35].

The peaks in the low molecular weight fraction were attributed to small oligosaccharides (Figures S1 and S2). The oligosaccharides were predominant in the extracts with a percentage from 48.70% to 93.06% in the pomace, and from 32.31% to 86.19% in the marc (Table 2). Doco et al. [36] indicated that these oligosaccharides correspond to xyloglucan oligosaccharides from the exocarp and mesocarp of grape berry cell wall material, as well as oligomers of homogalacturonans. Homogalacturonans are important pectic polysaccharides in grapes and consist of linear chains of D-galacturonic acid with α-(1–4) linkages. Another abundant molecule in the grape cell wall is RG-I, with an average molecular weight of around 40 kg/mol. However, these polysaccharides are difficult to extract, and they are insoluble [34].

Most of the doses of the different enzymes, in both ratios, partially degraded PRAG, MAN and TSP Non Pec fragments with molecular weights ranging from 400 kDa to 20 kDa in percentage higher than the control marc. However, the extraction of fragments in the pomace depended on the ratio used. Thus, in the lower ratio, only the highest dose of E1 achieved a higher extraction than the control; and in the higher ratio, most of the doses of E1 yielded higher release than the control (Table 2).

It should be noted that the highest dose of E1 was the only one that released a higher percentage of high molecular weight polysaccharides, in both ratios in the pomace extracts, and in the lower ratio in the marc (Table 2). In Section 2.1, it was mentioned that E1.4 achieved the highest extraction of MAN and TSP Non Pec at the ratio 4/1 in the pomace extract. Table 1A also shows that the highest extraction of PRAG was obtained with E1.4 at the 1.3/1 ratio in the pomace extract. Probably, in the pomace extract, the high molecular weight PRAG fragments were predominant over the TSP Non Pec at the lower ratio, while the high molecular weight TSP Non Pec were the majority at the higher ratio. In the pomace extracts, for both ratios, the mean percentage of polysaccharides of high molecular weight (Mw) (PRAG or TSP Non Pec) was 29.08%, 17.1% for medium Mw polysaccharides, and 53.8% for oligosaccharides. In the marc, 68.53% corresponded to high Mw PRAG or TSP Non Pec, 19.21% to medium Mw polysaccharides, and 15.30% to oligomers (Table 2). These results indicated that the greater deconstruction of the cell wall of marc compared to pomace favored a greater release of high Mw polysaccharides by the hydrolytic activity of E1 at the maximum dose.

The highest percentage of medium Mw polysaccharides was found in the pomace extracted with the E1 enzyme at its highest dose and higher ratio (Table 2). The RG-II content was the highest in this extract (Section 2.1). However, the first two doses of El at the higher ratio and the maximum dose of E1 at the lower ratio showed similar % of polysaccharides of medium Mw in marc. These results showed that the higher L/S ratio favored the extraction of medium Mw polysaccharides in marc and pomace, using low doses of E1 in marc, and the highest dose in pomace.

In the pomace, except for E1.4 and E4.4, the lower L/S ratio of all the enzymes released higher percentage of low Mw polysaccharides than the control (Table 2). At the lower ratio, E2.2 and the highest dose of E2 released 90% of low Mw polysaccharides. This result partially agreed with data shown in Section 2.1, where the E2 enzyme at both ratios released a higher HL content than the control. However, no significant differences were found in the TMS, RG-II, MAN, PRAG, TSP Pec, TSP Non Pec, and TSP content. Probably, the extraction of small oligosaccharides was higher in lower solvent volume in the pomace. More studies need to be done on this subject. Another possible explanation is that chemical degradation (due to the acidic solvent pH) predominantly released small oligosaccharides in the marc control at both ratios and in the pomace control at the higher ratio. In contrast, enzymatic activity at most doses partially degraded high and medium Mw polysaccharides in the marc extracts at both ratios and in the pomace extracts at higher ratios compared to the controls.

The pomace extract treated with E1 at the maximum dose showed a sum of percentage of high and medium Mw polysaccharides of 41.09% at the lower ratio, and 51.3% at the higher ratio. In contrast, the marc extract treated with E1 at the lower ratio contained 84.74%. In these pomace extracts (E1.4), 46.19% of the polysaccharides corresponded to high and medium Mw PRAG, TSP Non Pec, or RG-II, while 53.80% were oligomers. The combined percentage of high and medium Mw PRAG, TSP Non Pec, or RG-II was 1.9 times higher in the marc extract than in the pomace extracts, whereas the percentage of oligomers was 3.52 times lower than in the pomace extracts. Therefore, pomace extracts obtained by E1.4 at both ratios, and marc extracts obtained by E1.4 at the lower ratio, could be selected as potential additives in wine due to their higher content of pectic and non-pectic families of high and medium Mw. Chong et al. [37] concluded that a mixture of rhamnogalacturonan I/II, arabinan, arabinogalactan types I and II, and xyloglucan from grapes were key determinants of overall mouthfeel descriptors, particularly viscosity, astringency, and roughness. In contrast, homogalacturonans were clearly negatively correlated with mouthfeel and positively correlated with acidity in wines. There is limited information in the literature regarding the influence of grape oligosaccharides on the physicochemical and sensory properties of the wines. Canalejo et al. [7] indicated that oligosaccharides and polysaccharides of medium Mw interacted to a greater extent with the aromatic compounds of *Vitis vinifera* L. Viura white wine than polysaccharides of high Mw. In this sense, pomace extracts treated with E2 at the highest and second dose and the lower ratio would be selected as a potential additive in wine to obtain a higher proportion of oligosaccharides.

### 2.3. Purity of Polysaccharides and Extraction Efficiency

**Figure 1:**
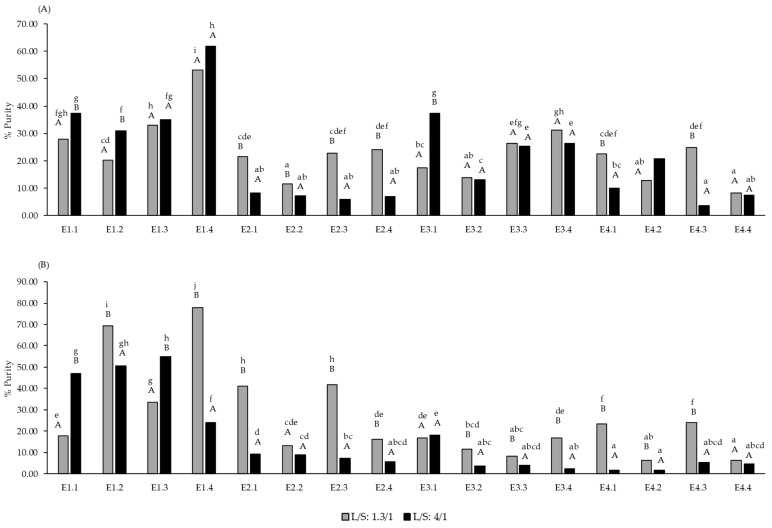
Purity of the extracts (% *w*/*w*, g total soluble polysaccharides/100 g extract) in white grape pomace (**A**) and red grape marc (**B**) from extractions assisted by the enzymatic doses E1, E2, E3 and E4 at the L/S ratio of 1.3/1 and 4/1. Different letters indicate statistical differences (*p* < 0.05). Lowercase letters compare the same parameter at the same liquid/solid ratio. Uppercase letters compare the same parameter at different liquid/solid ratios (*n* = 3).

**Figure 2:**
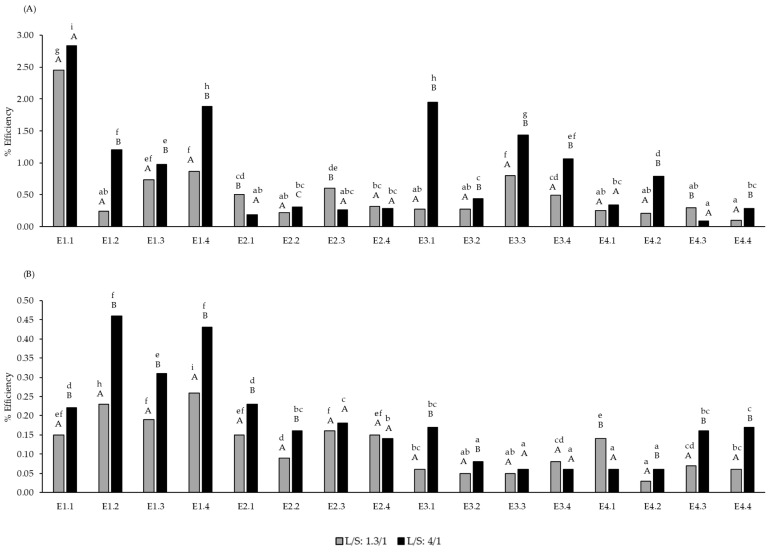
Extraction efficiency (% *w*/*w*, g total soluble polysaccharides/100 g dried pomace or marc) in white grape pomace (**A**) and red grape marc (**B**) from extractions assisted by the enzymatic doses E1, E2, E3 and E4 at the L/S ratio of 1.3/1 and 4/1. Different letters indicate statistical differences (*p* < 0.05). Lowercase letters compare the same parameter at the same liquid/solid ratio. Uppercase letters compare the same parameter at different liquid/solid ratios (*n* = 3).

The pomace extracts with the highest purity of total polysaccharides were obtained with E1 at the highest dose in both ratios, while the marc extracts with highest PS purity were obtained with the highest dose of E1 at the lower L/S ratio, and with doses 2 and 3 of E1 at the higher L/S ratio (Figure 1). The purity values, which ranged from 50% to 79%, were similar to the results reported by Canalejo et al. [8] using flash extraction techniques. However, the extraction efficiency percentages of total polysaccharides were low for the pomace and even lower for the marc. Probably, the short time that the pomace/marc was in contact with the enzymes in this study (24 h) could explain the lack of enzyme efficiency. Other authors have obtained higher efficiency values by employing different analytical procedures [38]. However, it should be noted that these methods utilize grape skins rather than grape pomace, which also includes remnants of pulp, seeds, and stems. Also, Minjares-Fuentes et al. [39] recovered 32% of pectin from grape pomace using an ultrasound-assisted procedure. The highest efficiency percentage values were obtained in the pomace with the minimum dose of E1 in both ratios (Figure 2). These results indicated that the enzyme E1 at low doses with 4/1 ratio and at maximum doses (regardless of the ratio) can be combined with other green extraction techniques, such as ultrasound or microwave, among others, to increase the efficiency of polysaccharide extraction from pomace/marc and the purity of the extracts.

## 3. Materials and Methods

### 3.1. Grape Pomace Materials and Enological Parameters

Samples of pomace from *Vitis vinifera* L. Viura and marc from *Vitis vinifera* L. Tempranillo tinto were collected from a winery in the Rioja Alta subregion from the 2023 harvest. Both white and red grapes were harvested at the point of ripeness decided by the winemaker. The white grapes were then sulfited (45 mg/L of total SO_2_) and pressed in a pneumatic press. The red grapes were crushed, destemmed, and sulfited (45 mg/L of total SO_2_). After fermentation–maceration, the solid part was pressed in a vertical hydraulic press. The pressed white grape pomace and the red grape marc were immediately frozen at −15 °C. The dry weight of the *Vitis vinifera* L. Viura pomace was 52.44% *w*/*w*, and of the *Vitis vinifera* L. Tempranillo marc, 44.5% *w*/*w*, in good accordance with values reported in the literature [40].

Classical oenological parameters of the grapes were determined according to the official OIV methods [41]. The probable alcohol content of the *Vitis vinifera* L. Viura must was 12.4% *v*/*v*, and for the *Vitis vinifera* L. Tempranillo, 14.6% *v*/*v*. The pH was 3.4 for both musts, with a total acidity of 4.6 and 5.4 g/L H_2_T, respectively.

### 3.2. Assays of Extraction Assisted by Commercial Enzymes and Obtention of Polysaccharide Extracts from Grape Pomace and Marc

Marc from *Vitis vinifera* L. Tempranillo tinto and pomace from *Vitis vinifera* L. Viura, once defrosted, were treated following the procedure described by Cano-González et al. [42], with some modifications. The marc and pomace were dried in an air oven at 40 °C for 48 h, crushed with a Thermomix TM 6 (Vorwerk, Bilbao, Spain) and then sieved to a particle size < 555 µm.

The enzymes used were supplied by Agrovin, S.A. (Ciudad Real, Spain). Table 3 shows the nomenclature of the enzymes and the enzymatic activities of each enzyme. Enzymes E1, E3 and E4 showed different polygalacturonase, pectin lyase and pectin methyl esterase activities (U/g). In addition, E3 and E4 showed β-glucanase activity and E3 also arabinase activity. Only the E2 enzyme showed polygalacturonase + pectin lyase activity (Table 3).

The minimum enzyme dose used was double that recommended by Agrovin S.A. (Ciudad Real, Spain). From the minimum dose, a dose scale was made and the L/S ratio used (L/S = 1.3/1 and L/S = 4/1) was considered to calculate the amount of enzyme added to each test (Table 4). The L/S ratio of 4/1 was selected because of the high purity results (mg total polysaccharides/g extract) obtained by our group using the flash extraction technique [8]. To save solvent, it was considered of interest to test the L/S ratio of 1.3/1. The solvent used was tartaric acid (TH_2_) at 2.5 g/L and pH 3.2.

Enzyme-assisted extractions (EAE) and the control assay (without enzyme addition) were carried out in an incubator (Refrigerated Incubator Shaker, Innova 4330, Marshall Scientific, Hampton, VA, USA) for 24 h under agitation (150 rpm) at 20 °C. Extractions were performed in triplicate. Thereafter, the samples were centrifuged (13789 RCF, Sorvall Lynx 4000 Centrifuge, Thermo Scientific, Madrid, Spain). The supernatants obtained were concentrated five times using a rotary evaporator (Rotavapor R-200, Büchi, Büchi Ibérica S.L.U., Barcelona, Spain), as described by Canalejo et al. [8]. Polysaccharides from the concentrated liquid were recovered by precipitation after the addition of ethanol acid as previously described [8,30]. The precipitates were centrifuged (13789 RCF, Sorvall Lynx 4000 Centrifuge, Thermo Scientific), and the sediments were lyophilized. The freeze-dried sediments were considered as the polysaccharide extracts from the marc and pomace by EAE.

### 3.3. Identification and Quantification of Monosaccharides by GC-MS and Quantification of Polysaccharide Families

GC was performed on an Agilent 7890A gas chromatograph (Agilent Technologies, Waldbronn, Germany) coupled to a 5975C VL quadrupole mass detector (MS).

The chromatographic column was a Teknokroma (Barcelona, Spain) fused silica capillary column (30 m × 0.25 mm × 0.25 mm) of phase 5% phenyl/95% methyl polysiloxane. The oven program started at an initial temperature of 120 °C which was increased at a rate of 1 °C/min to 145 °C and then to 180 °C at a rate of 0.9 °C/min, and finally to 230 °C at 40 °C/min. The GC injectors were equipped with a 3.4 mm I.D. liner and were maintained at 250 °C with a 1:20 split ratio. The carrier gas was helium (99.996%) at a flow rate of 1 mL/min. Ionization was performed by electron impact (EI) mode at 70 eV. The temperatures used were 150 °C for the MS Quad, 230 °C for the MS Source, and 250 °C for the transfer line.

The monosaccharide composition was determined by GC–MS of their trimethylsilyl-ester *O*-methyl glycosyl residues obtained after acidic methanolysis and derivatization as previously described [8,30]. A quantity of 100 μL of myo-inositol (1 mg/mL) (Fluka, Buch, Switzerland) was added to the extracts as internal standard, and freeze-dried. Thereafter, they were treated with 1 mL of the methanolysis reagent [MeOH anhydrous (Merck, Darmstadt, Germany) containing CH_3_COCl 0.5 M], and the reaction was conducted in nitrogen atmosphere at 80 °C for 16 h. After removing the excess of reagent with a stream of nitrogen, the conversion of the methyl glycosides to their trimethylsilyl derivates was performed by adding 0.5 mL of a mix of pyridine/hexamethyldisilazane/trimethylchlorosilane (10:2:1 *v*/*v*) (Merck, Darmstadt, Germany). The reaction was carried out at 80 °C for 30 min and the reagent was removed using a stream of nitrogen gas. Finally, the derivatized residues were extracted with 1 mL of hexane (Merck, Darmstadt, Germany). Samples of polysaccharide extracts from the derivatized marc and pomace were analyzed in triplicate and standard carbohydrates were used for identification quantification [8,30].

The total monosaccharides components of the polysaccharide extracts were called TMS. The results of the monosaccharide composition (mg monosaccharide/g extract) of *Vitis vinifera* L. Viura pomace and *Vitis vinifera* L. Tempranillo marc from the extractions assisted by enzyme doses E1, E2, E3, and E4 and control of *Vitis vinifera* L. Viura pomace and *Vitis vinifera* L. Tempranillo marc, at L/S ratio of 1.3/1 and 4/1, are presented in Table S1 and Table S2, respectively.

The different pectic polysaccharide families were calculated as described [30]. Polysaccharides rich in arabinose and galactose (PRAG) were estimated from the sum of galactosyl, arabinosyl, rhamnosyl and glucuronosyl residues; the rhamnogalacturonan-II (RG-II) content was calculated from the sum of its diagnostic monosaccharides 2-*O*-methyl fucose, 2-*O*-methyl xylose, apiose and 3-deoxyoctulosonic acid which represent approximately 25% of the RG-II molecule. Considering the molar ratios of RG-II (1 residue of 2-*O*-methyl fucose, 3.5 of rhamnose, 2 of arabinose, 2 of galactose, 1 of glucuronic acid and 9 of galacturonic acid), the remaining part was attributed to the presence of PRAG in the case of rhamnose, arabinose, galactose and glucuronic acid. The remaining galacturonosyl residues were used to estimate the homogalacturonan (HL) content. Glucose and xylose content was attributed to non-pectic polysaccharides and mannose (MAN) content to mannans and hemicellulose, considered also as non-pectic polysaccharides. The sum of PRAG, RG-II and HL was expressed as total soluble pectic polysaccharides (TSP Pec) and the sum of mannose, glucose and xylose as total soluble non-pectic polysaccharides (TSP Non Pec). The sum of TSP Pec and TSP Non Pec was expressed as the total soluble polysaccharides (TSP).

### 3.4. Polysaccharide Analysis by HPSEC-RID

The molecular weights and molecular weight distributions of the grape polysaccharides from the EAE extracts were determined following the method previously described [30].

Samples were analyzed using high-performance size-exclusion chromatography with a refractive index detector (HPSEC-RID) (1100 Agilent liquid chromatograph, Agilent Technologies, Waldbronn, Germany, equipped with one G1311A quaternary pump, an on-line G1379A degasser, a G1316A column oven, a G1362 refractive index detector, and a G1313A automatic injector). Two serial Shodex OHpack KB-803 and KB-805 columns (0.8 × 30 cm, Showa Denko, Tokyo, Japan) were used. The freeze-dried extracts from the marc and pomace were solved in miliQ water (4.0 mg/mL), filtered through a membrane with a 0.45 µm pore size and 500 μL was injected and eluted with a 0.1 M solution of LiNO_3_ (Merck, Darmstadt, Germany) at a flow rate of 1 mL/min. The molecular weights of the different polysaccharide fractions were determined with narrow pullulan molecular weight standards (Shodex P-82, Waters, Barcelona, Spain): P-5, Mw = 5.9 kDa; P-10, Mw = 11.8 ka; P-20, Mw = 22.8 kDa; P-50, Mw = 47.3 kDa; P-100, Mw = 112 kDa; P-200, Mw = 212 kDa; P-400, Mw = 404 kDa. The apparent molecular weights were deduced from the calibration equation log Mw = 10.387 − 0.3749 tR (tR = column retention time at peak maximum, and r^2^ = 0.998).

### 3.5. Polysaccharide Purity in Extracts and Enzyme-Assisted Extraction (EAE) Efficiency

The total soluble polysaccharide (TSP) purity of marc and pomace extracts was expressed in percent *w*/*w* (g TSP/100 g dried extract). The EAE efficiency of TSP was expressed in percent *w*/*w* (g TSP/100 g dried marc or pomace).

### 3.6. Statistical Analysis

A three-factor experimental design was carried out to evaluate the effect of enzyme type, enzyme dose and liquid/solid (L/S) ratio. For each of the varietal pomace or marc, three samples were analyzed for each combination of the four enzyme types evaluated, the four different dosage levels and the two different L/S ratios. A control level (without enzymes) combined with the two L/S ratios was considered, also analyzing three samples for each combination. Therefore, this design contemplated a total of 34 experimental conditions: (4 enzyme types × 4 dose levels + 1 control) × 2 L/S levels.

Statistical analysis was performed using the SPSS v. 15.0 for Windows statistical package (SPSS Statistics, Inc., Chicago, IL, USA). A one-way analysis of variance (one-way ANOVA) was applied to determine significant differences among enzymes, liquid/solid ratios, and enzyme dosages to identify the optimal extraction conditions. Results were considered statistically significant at *p* < 0.05.

## 4. Conclusions

The deconstruction of the cell wall of pomace and marc by EAE depends on the mixture of enzyme activities that make up the commercial enzyme, the dose and the L/S ratio. The highest dose of the commercial enzyme E1, composed of polygalacturonase, pectin lyase and pectin-methyl-esterase (with the highest activity), proved to be the most effective in the degradation of high and medium molecular weight pectic families (PRAG, RG-II) and non-pectic (MAN and TSP Non Pec) of pomace/marc. In the low L/S ratio, the difference in the deconstruction of the cell wall between marc and pomace explains the fact that the highest doses of E1 degrade high and medium molecular weight pectic and non-pectic polysaccharides in marc. Therefore, the extracts obtained with these conditions could be selected as potential additives in wine due to their higher content of RG II + PRAG + MAN + TSP Non Pec of high and medium molecular weight, as they are key families in the mouthfeel descriptors of wine. The effects of the solid–liquid ratio on polysaccharide extraction from pomace/marc were not clear, as they varied depending on the substrate (pomace or marc), the type of commercial enzyme and the dose. More studies should be carried out on this topic.

The highest purity of total polysaccharides in the pomace extracts was obtained with the highest dose of E1 in both L/S ratios, and in the marc at the lower L/S ratio. At the higher S/L ratio, doses 2 and 3 of E1 achieved high values of purity of total polysaccharides in the marc.

The efficiency of total polysaccharide extraction from pomace/marc by the enzymes used was low. Enzyme E1 at low doses and at the maximum dose (regardless of the S/L ratio) could be combined with other green extraction techniques to increase the efficiency of polysaccharide extraction from pomace/marc and the purity of the extracts.

## Figures and Tables

**Table 1:** (**A**) Concentration of total monosaccharides (mg TMS^a^/g extract) and of polysaccharide families (mg polysaccharide family/g extract) of *Vitis vinifera* L. Viura pomace from the extractions assisted by enzymes doses E1, E2, E3, and E4 at L/S ratios of 1.3/1 and 4/1. (**B**) Concentration of total monosaccharides (mg TMS^a^/g extract) and of polysaccharide families (mg polysaccharide family/g extract) of *Vitis vinifera* L. Tempranillo marc from the extractions assisted by enzymes doses E1, E2, E3, and E4 at L/S ratios of 1.3/1 and 4/1.

**(A)**																
**Sample ^b^**	**L/S: 1.3/1**								**L/S: 4/1**							
	**TMS ^a^**	**RG-II ^a^**	**MAN ^a^**	**PRAG ^a^**	**HL ^a^**	**TSP Pec ^a^**	**TSP Non Pec ^a^**	**TSP ^a^**	**TMS ^a^**	**RG-II ^a^**	**MAN ^a^**	**PRAG ^a^**	**HL ^a^**	**TSP Pec ^a^**	**TSP Non Pec ^a^**	**TSP ^a^**
Control	260.02 ab β	45.71 a α	56.03 a β	79.02 ab α	14.21 a α	138.94 ab α	125.56 a β	264.50 ab β	130.05 a α	26.39 a α	16.65 a α	52.26 a α	6.44 a α	85.09 a α	47.45 a α	132.54 a α
E1.1	288.37 b B α	71.92 ab B α	18.84 a BC α	105.48 bc B β	41.56 ab BC α	218.96 bc C α	61.08 a A α	280.04 ab B α	388.67 c B β	173.25 b D β	11.59 a A α	70.38 b B α	42.46 c AB α	286.10 b B β	88.00 b B β	374.10 c B β
E1.2	203.83 a B α	21.29 a A α	4.60 a A α	47.48 a B α	45.46 ab C β	114.23 a B α	87.02 a C β	201.25 a B α	309.71 b D β	141.31 b B β	17.01 a B β	83.49 bc B β	28.61 b B α	253.41 b D β	56.28 a C α	309.69 b D β
E1.3	372.50 c B α	82.34 ab B α	10.17 a A α	137.02 c C α	33.66 a B α	253.02 c C α	77.06 a A α	330.08 b B α	356.78 c C α	152.79 b B β	16.97 a B β	113.11 d C α	31.71 bc B α	297.61 b C β	53.09 a B α	350.70 bc C α
E1.4	542.71 d C α	135.17 b B α	21.29 a B α	227.44 d B β	82.37 c B α	444.98 d C α	87.35 a B α	532.33 c D α	627.08 d C α	417.17 c B β	31.63 b B α	94.77 c B α	0.00 a A α	511.94 c C α	105.34 c B α	617.27 d C α
Control	260.02 ab β	45.71 a α	56.03 a β	79.02 ab α	14.21 a α	138.94 ab α	125.56 a β	264.50 ab β	130.05 a α	26.39 a α	16.65 a α	52.26 a α	6.44 a α	85.09 a α	47.45 a α	132.54 a α
E2.1	217.12 b A β	18.07 a A α	4.34 a A α	57.76 ab A β	51.02 c C β	126.86 a ABβ	87.90 a B β	214.76 b A β	86.86 a A α	24.87 a B β	8.57 a A	27.33 a A α	4.56 ab A α	56.76 ab A α	26.31 a A α	83.07 a A α
E2.2	119.82 a A β	22.11 a A α	7.75 a A α	14.81 a A α	16.91 ab B β	53.83 a A α	60.54 a B β	114.37 a A β	77.40 a A α	29.89 a A	7.02 a A α	19.48 a A α	0.15 a A α	49.52 a A α	23.25 a A α	72.77 a A α
E2.3	231.39 b A β	31.37 a A β	9.89 a A α	44.47 ab A β	31.09 b B β	106.92 a A β	121.76 a B β	228.68 b A β	61.03 a A α	3.53 a A α	7.46 a A α	19.11 a A α	11.45 b A α	34.09 a A α	24.85 a A α	58.95 a A α
E2.4	242.78 b B β	23.55 a A α	31.67 a C β	79.42 b A β	7.41 a A β	110.38 A β	130.05 a C β	240.43 b B β	73.86 a A α	25.99 a A α	7.56 a A	17.78 a A α	2.03 a A α	45.79 a A α	23.86 a A α	69.65 a A α
Control	260.02 ab β	45.71 a α	56.03 a β	79.02 ab α	14.21 a α	138.94 ab α	125.56 a β	264.50 ab β	130.05 a α	26.39 a α	16.65 a α	52.26 a α	6.44 a α	85.09 a α	47.45 a α	132.54 a α
E3.1	174.23 a A α	22.44 a A α	24.22 a C α	52.95 a A α	30.65 b B α	106.05 a A α	68.70 a A α	174.75 a A α	392.45 c B β	135.64 b C β	36.87 b B α	87.23 c B β	61.69 bc B α	284.56 c B β	89.53 b B α	374.09 c B β
E3.2	122.23 a A α	59.31 a C β	14.79 a B α	42.90 a B α	0.00 a A α	102.21 a B α	36.62 a A α	138.82 a A α	134.70 a B α	25.59 a A α	16.17 a B α	35.12 a A α	32.14 ab B β	92.85 a B α	38.81 a B α	131.66 a B α
E3.3	267.46 b A α	79.50 a B β	21.62 a B α	75.30 a AB α	40.25 b B α	195.05 ab B α	68.57 a A α	263.62 b ABα	255.90 b B α	16.31 a A α	31.38 b C β	70.06 c B α	69.89 c C β	156.26 b B α	97.59 b C β	253.85 b B α
E3.4	272.38 b A α	173.21 b C β	34.02 a C α	56.74 a A α	0.00 a A α	229.95 b B α	81.03 a B α	310.98 b C α	265.81 b B α	13.92 a A α	37.83 b B α	86.08 c B α	71.35 c B β	171.35 b B α	92.48 b B α	263.83 b B α
Control	260.02 ab β	45.71 a α	56.03 a β	79.02 ab α	14.21 a α	138.94 ab α	125.56 a β	264.50 ab β	130.05 a α	26.39 a α	16.65 a α	52.26 a α	6.44 a α	85.09 a α	47.45 a α	132.54 a α
E4.1	222.02 b A β	68.95 a B β	14.79 a B α	95.11 b B β	0.00 a A α	164.07 b B β	61.51 ab A β	225.58 b AB β	101.63 bc Aα	7.72 a A α	14.49 bc A α	41.60 b A α	8.71 b A β	58.03 bc A α	41.35 b A α	99.39 bc A α
E4.2	131.05 a A α	36.05 a B β	11.49 a B α	50.00 ab B α	2.49 ab A α	88.53 ab B α	38.89 ab A α	127.42 a A α	208.47 d C β	16.02 a A α	33.43 d C β	91.48 c B β	7.02 b A β	114.53 d C β	91.93 c D β	206.47 d C β
E4.3	247.63 b A β	89.19 a B β	18.79 a B β	88.85 b B β	0.00 a A α	178.05 b B β	70.74 ab A β	248.79 b A β	40.48 a A α	1.54 a A α	6.82 a A α	11.45 a A α	5.97 ab A β	18.95 a A α	17.66 a A α	36.61 a A α
E4.4	83.06 a A α	28.67 a A α	9.65 a A α	28.66 a A β	0.00 a A α	57.34 a A α	25.40 a A α	82.74 a A α	73.27 b A α	26.24 a A α	9.17 ab A α	19.30 a A α	0.00 a A α	45.54 ab A α	28.79 a A α	74.33 b A α
**(B)**																
**Sample ^b^**	**L/S: 1.3/1**								**L/S: 4/1**							
	**TMS ^a^**	**RG-II ^a^**	**MAN ^a^**	**PRAG ^a^**	**HL ^a^**	**TSP Pec ^a^**	**TSP Non Pec ^a^**	**TSP ^a^**	**TMS ^a^**	**RG-II ^a^**	**MAN ^a^**	**PRAG ^a^**	**HL ^a^**	**TSP Pec ^a^**	**TSP Non Pec ^a^**	**TSP ^a^**
Control	55.48 a α	11.22 a β	8.80 a α	23.78 a α	1.40 a α	36.40 a β	17.81 a α	54.21 a α	44.26 a α	5.32 a α	7.94 a α	18.45 a α	3.31 a α	27.08 a α	14.72 a α	41.80 a α
E1.1	177.79 b A α	47.63 b C α	23.05 b A α	57.23 b A α	18.67 b A α	123.54 b A α	54.50 b A α	178.04 b A α	472.76 c D β	160.01 c C β	65.10 c D β	100.97 c D β	76.52 bc B α	337.51 c D β	132.31 c C β	469.82 c D β
E1.2	714.16 d B β	243.20 d B α	91.90 d B β	205.05 d C β	85.86 d B β	534.11 d B β	157.63 d C β	691.74 d C β	524.59 cd C α	230.89 d B α	62.27 c C α	110.02 c D α	40.73 ab B α	381.65 cd C α	124.06 c D α	505.70 cd C α
E1.3	337.82 c C α	86.72 c B α	49.40 c B α	102.82 c B α	61.35 c B α	250.89 c C α	83.42 c C α	334.31 c C α	570.84 d B β	218.22 d B β	77.47 d B β	103.36 c B α	88.88 c B α	410.47 d B β	138.82 c B β	549.29 d B β
E1.4	792.77 e C β	240.38 d C β	109.59 e C β	227.22 e C β	94.60 d D β	562.19 d C β	217.91 e B β	780.10 e C β	252.29 b C α	124.19 b C α	23.57 b C α	40.29 b C α	21.32 b C α	185.80 b C α	55.10 b C α	240.90 b C α
Control	55.48 a α	11.22 a β	8.80 a α	23.78 a α	1.40 a α	36.40 a β	17.81 a α	54.21 a α	44.26 a α	5.32 a α	7.94 a α	18.45 a α	3.31 a α	27.08 a α	14.72 a α	41.80 a α
E2.1	407.19 c C β	104.88 c D β	49.22 c C β	145.35 c C β	36.38 c AB β	286.61 d C β	124.70 c B β	411.31 c C β	93.67 c B α	20.19 b AB α	13.07 bc B α	38.73 b B α	5.83 b A α	64.74 c B α	27.72 b B α	92.47 c B α
E2.2	133.14 b A α	30.35 b A β	15.58 b A α	47.27 b B α	12.78 ab A α	90.40 b A α	42.39 b B α	132.79 b B α	91.22 bc B α	7.70 a A α	14.21 c B α	41.95 b C α	13.44 c A α	63.09 c B α	27.05 b C α	90.14 c B α
E2.3	411.06 c C β	109.72 c C β	74.91 d C β	182.96 d C β	0.00 a A α	292.68 d C β	124.71 c D β	417.40 c D β	74.10 b A α	21.08 b A α	9.79 ab A α	26.23 a A α	2.58 ab A	49.89 b A α	22.89 ab A α	72.78 bc A α
E2.4	165.85 b B β	36.40 b B β	19.24 b AB β	59.80 b B β	26.28 bc B β	122.48 c B β	38.22 b A β	160.70 b B β	55.02 a B α	18.45 b B α	10.20 abc B α	18.59 a B α	0.00 a A α	37.04 a B α	18.18 a B α	55.22 ab B α
Control	55.48 a α	11.22 a β	8.80 a α	23.78 a α	1.40 a α	36.40 a β	17.81 a α	54.21 a α	44.26 a α	5.32 a α	7.94 a α	18.45 a α	3.31 a α	27.08 a α	14.72 a α	41.80 a α
E3.1	170.31 c A α	14.83 ab A α	24.10 b A α	70.42 c AB α	43.48 d AB α	128.73 c A α	39.92 c A α	168.65 c A α	185.07 c C α	31.22 b B β	27.19 c C α	66.09 c C α	40.55 c AB α	137.87 b C α	43.24 b B α	181.10 b C α
E3.2	118.13 b A β	28.10 c A β	13.13 a A β	37.39 ab AB β	22.18 b A β	87.67 b A β	28.31 b A β	115.99 b AB β	37.25 ab A α	2.87 a A α	7.95 ab AB α	16.06 b B α	2.13 a A α	21.06 a A α	15.05 a B α	36.11 a A α
E3.3	82.13 a A β	19.00 b A β	13.13 a A α	36.53 a A β	0.00 a A α	55.53 a A β	27.16 b A β	82.69 a A β	42.18 ab A α	3.44 a A α	9.09 b A α	18.51 b A α	2.91 a A β	24.86 a A α	15.33 a A α	40.19 a A α
E3.4	172.50 c B β	42.96 d B β	22.56 b B β	50.65 b B β	35.23 c C β	128.85 c B β	38.99 c A β	167.83 c B β	25.54 a A α	1.12 a A α	4.31 a A α	8.40 a A α	7.60 b B α	17.12 a A α	8.06 a A α	25.19 a A α
Control	55.48 a α	11.22 a β	8.80 a α	23.78 a α	1.40 a α	36.40 a β	17.81 a α	54.21 a α	44.26 a α	5.32 a α	7.94 a α	18.45 a α	3.31 a α	27.08 a α	14.72 a α	41.80 a α
E4.1	235.98 b B β	29.70 b B β	35.75 b B β	85.24 b B β	60.30 d B β	175.24 b B β	58.32 b A β	233.56 b B β	17.84 a A α	4.24 a A α	3.40 a A α	7.19 a A α	0.00 a A α	11.43 a A α	6.23 a A α	17.66 a A α
E4.2	66.15 a A β	8.44 a A β	10.21 a A β	22.77 a A β	12.52 b A β	43.72 a A β	20.54 a A β	64.26 a A β	18.04 a A α	0.78 a A α	3.02 a A α	6.19 a A α	4.63 b A α	11.60 a A α	5.51 a A α	17.11 a A α
E4.3	241.14 b B β	31.87 b A β	40.60 b B β	97.49 b B β	44.65 c B β	174.00 b B β	65.43 b B β	239.43 b B β	52.97 b A α	1.90 a A α	8.16 b A α	17.37 b A α	17.03 d A α	36.30 b A α	15.49 b A α	51.79 b A α
E4.4	63.34 a A α	8.21 a A β	8.09 a A α	21.21 a A α	15.07 b A α	44.49 a A β	17.14 a A α	61.64 a A α	51.20 b B α	3.59 a A α	6.66 ab A α	20.08 b B α	8.26 c B α	31.94 b B α	16.39 b B α	48.32 b B α

^a^ TMS: total monosaccharides; RG-II: rhamnogalacturonan type II; MAN: mannose; PRAG: polysaccharides rich in arabinose and galactose; HL: homogalacturonans; TSP Pec: total pectic polysaccharides; TSP Non Pec: total non-pectic polysaccharides; TSP: total soluble polysaccharides. ^b^ See nomenclature in Section 3.2. Different letters indicate statistical differences (*p* < 0.05). Lowercase letters compare different doses of the same enzyme. Capital letters compare equal doses of different enzymes. Greek letters compare different ratios (*n* = 3).

**Table 2:** Percentage of molecular weight distribution of *Vitis vinifera* L. Viura pomace (**A**) and *Vitis vinifera* L. Tempranillo marc (**B**) from extractions assisted by the enzymatic doses E1, E2, E3 and E4 in the L/S ratio of 1.3/1 and 4/1.

(A)	L/S = 1.3/1			L/S = 4/1				(B)	L/S = 1.3/1			L/S = 4/1		
Pomace	% HMW ^a^	% MMW	% LMW	% HMW	% MMW	% LMW		Marc	% HMW	% MMW	% LMW	% HMW	% MMW	% LMW
Control	20.91	9.93	69.16	11.54	8.74	79.73		Control	11.90	3.32	84.78	8.95	6.68	84.37
E 1.1	11.86	4.84	83.30	17.25	13.37	69.38		E 1.1	16.78	6.29	76.93	48.30	19.39	32.31
E 1.2	12.79	7.15	80.06	17.76	14.69	67.55		E 1.2	26.28	11.00	63.00	47.76	18.66	33.57
E 1.3	14.26	7.99	77.76	17.80	15.10	67.10		E 1.3	23.53	2.50	74.25	47.36	17.72	35.00
E 1.4	31.61	9.48	58.91	26.55	24.75	48.70		E 1.4	68.53	19.21	15.30	23.21	16.89	59.90
E 2.1	10.79	2.48	86.73	14.04	9.45	76.51		E 2.1	40.12	8.88	51.66	10.76	5.47	83.77
E 2.2	5.19	4.14	90.67	13.60	12.04	74.36		E 2.2	12.78	7.10	80.36	15.88	6.89	77.22
E 2.3	8.68	6.83	84.49	11.33	10.14	78.53		E 2.3	45.61	9.72	44.73	13.55	8.57	77.88
E 2.4	4.54	2.41	93.06	9.26	5.49	85.26		E 2.4	16.27	8.73	75.00	9.54	4.27	86.19
E 3.1	11.79	2.76	85.45	15.08	9.45	75.47		E 3.1	27.24	1.45	71.31	20.75	10.85	68.40
E 3.2	13.81	8.59	77.60	14.90	9.72	75.38		E 3.2	17.67	2.45	79.88	12.77	8.01	79.23
E 3.3	9.94	3.68	86.38	14.35	11.37	74.28		E 3.3	8.46	6.44	85.10	14.65	9.60	75.75
E 3.4	14.59	11.70	73.72	15.40	7.33	77.27		E 3.4	20.11	12.00	67.89	9.64	6.78	83.58
E 4.1	7.47	5.92	86.60	15.06	9.48	75.46		E 4.1	27.67	9.78	62.55	11.20	10.76	78.04
E 4.2	11.67	9.08	79.25	16.58	9.43	74.00		E 4.2	14.44	4.80	80.76	16.73	14.50	69.22
E 4.3	20.15	6.74	73.11	11.73	6.96	81.31		E 4.3	35.35	10.59	54.09	13.49	9.09	77.42
E 4.4	19.12	11.27	69.61	11.80	7.82	80.38		E 4.4	17.13	3.20	79.67	16.25	11.74	72.01

^a^ HMW: high molecular weight fraction (20–400 kDa); MMW: medium molecular weight fraction (5.9–20 kDa); LMW: low molecular weight fraction (<5.9 kDa).

**Table 3:** Nomenclature and enzymatic activities of the commercial enzymes used in the assays.

		Enzymatic Activities (U/g)				
Enzyme	Commercial Name	Polygalacturanase	Pectinlyase	Pectin Methyl Esterase	Arabinase	β-Glucanase
E1	Enozym lux	4500	130	1000	-	-
E2	-	7800	200	-	-	-
E3	-	5300	450	560	90	2400
E4	Enozym vintage	3704	280	450	-	3608

**Table 4:** Dose of enzymes (μL E/mL) and amount of enzyme (μL E/g of pomace or marc) according to the liquid/solid ratio (L/S).

Nomenclature of Doses	Nomenclature of Enzyme and Dose ^a^	Dose of Enzyme	L/S: 1.3/1	L/S: 4/1
		μL E/mL	μL E/g Marc or Pomace	μL E/g Marc or Pomace
1	E1.1; E2.1; E3.1 and E4.1	0.02	0.026	0.08
2	E1.2; E2.2; E3.2 and E4.2	0.03	0.039	0.12
3	E1.3; E2.3; E3.3 and E4.3	0.08	0.104	0.32
4	E1.4; E2.4; E3.4 and E4.4	0.20	0.260	0.80

^a^ E1, E2; E3 and E4 are different enzymes while the doses are 1, 2, 3, 4 added after the code relating to the type of enzyme.

## Data Availability

The original contributions presented in this study are included in the article/Supplementary Materials. Further inquiries can be directed to the corresponding author.

## Supplementary Materials

The following supporting information can be downloaded at: https://www.mdpi.com/article/10.3390/molecules30020213/s1, Table S1: Monosaccharide composition (mg monosaccharide/g extract) of *Vitis vinifera* L. Viura pomace from the extractions assisted by enzymes doses E1, E2, E3, and E4 and control of *Vitis vinifera* L. Viura pomace at L/S ratio of 1.3/1 and 4/1; Table S2: Monosaccharide composition (mg monosaccharide/g extract) of *Vitis vinifera* L. Tempranillo marc from the extractions assisted by enzymes doses E1, E2, E3, and E4 and control of *Vitis vinifera* L. Tempranillo marc at L/S ratio of 1.3/1 and 4/1; Figure S1: HPSEC-RID chromatograms of total soluble polysaccharides in the extracts from enzyme-assisted extraction (EAE) in *Vitis vinifera* L. Viura pomace compared to the control sample at liquid/solid ratios (L/S) of 1.3/1 and 4/1. Chromatograms obtained using two serial Shodex OHpack KB-803 and KB-805 columns. HMW: high molecular weight fraction (20–400 kDa); MMW: medium molecular weight fraction (5.9–20 kDa); LMW: low molecular weight fraction (<5.9 kDa); E1, E2, E3, and E4: See nomenclature in Section 3.2; Figure S2: HPSEC-RID chromatograms of total soluble polysaccharides in the extracts from enzyme-assisted extraction (EAE) in *Vitis vinifera* L. Tempranillo marc compared to the control samples at liquid/solid ratio (L/S) of 1.3/1 and 4/1. Chromatograms obtained using two serial Shodex OHpack KB-803 and KB-805 columns. HMW: high molecular weight fraction (20–400 kDa); MMW: medium molecular weight fraction (5.9–20 kDa); LMW: low molecular weight fraction (<5.9 kDa); E1, E2, E3, and E4: See nomenclature in Section 3.2.
