# Supplementary material for: Recovery of Polysaccharides from Red Grape Marc and White Grape Pomace by Degradation of Cell Walls by Enzymes with Different Activities
Source: Molecules. 2025 Jan 7;30(2):213. doi: 10.3390/molecules30020213 (PMC11767955; doi:10.3390/molecules30020213)

Supplementary Materials:

Table S1. Monosaccharide composition (mg monosaccharide/g extract) of *Vitis vinifera* L. Viura pomace from the extractions assisted by enzymes doses E1, E2, E3, and E4 and control of *Vitis vinifera* L. Viura pomace at L/S ratio of 1.3/1 and 4/1.

	Sample[b]	2-*O*-MeFuc	2-*O*-MeXyl	Api	Ara	Rha	Fuc	Xyl	Man	Gal	GalA	Glu	GluA	Kdo	TMS
L/S: 1.3/1	Control	2.48 ± 0.69	1.32 ± 0.28	1.09 ± 0.86	35.77 ± 30.03	18.58 ± 3.74	0.96 ± 0.73	17.51 ± 10.20	56.03 ± 49.21	28.95 ± 16.96	29.00 ± 5.47	52.02 ± 22.45	15.59 ± 3.74	0.71 ± 0.22	260.02 ± 57.40
	E1.1	3.99 ± 0.92	2.00 ± 0.39	1.92 ± 0.54	57.11 ± 8.63	29.83 ± 5.20	1.48 ± 0.32	10.37 ± 1.70	18.84 ± 5.80	39.29 ± 8.74	77.50 ± 10.84	31.87 ± 1.78	11.19 ± 2.89	2.98 ± 2.59	288.37 ± 47.88
	E1.2	1.14 ± 0.29	0.63 ± 0.19	0.66 ± 0.14	27.77 ± 5.54	11.96 ± 1.81	0.52 ± 0.12	4.87 ± 1.61	4.60 ± 0.61	13.92 ± 2.69	55.72 ± 12.54	77.54 ± 17.43	2.96 ± 0.13	1.52 ± 1.05	203.83 ± 36.50
	E1.3	4.60 ± 1.51	2.25 ± 0.51	0.78 ± 0.09	84.33 ± 18.00	45.55 ± 12.46	1.76 ± 0.52	12.63 ± 3.43	10.17 ± 3.09	33.25 ± 5.24	75.13 ± 5.81	54.25 ± 5.28	10.75 ± 6.58	37.02 ± 36.23	372.50 ± 7.79
	E1.4	7.46 ± 2.26	3.80 ± 0.93	3.19 ± 1.07	137.09 ± 34.05	66.76 ± 15.42	2.77 ± 0.64	17.99 ± 1.80	21.29 ± 5.90	71.35 ± 16.01	149.50 ± 48.80	48.08 ± 12.83	11.91 ± 2.90	1.51 ± 0.43	542.71 ± 55.56
	E2.1	0.93 ± 0.10	0.58 ± 0.14	1.92 ± 0.53	22.82 ± 6.32	13.61 ± 2.74	0.50 ± 0.12	6.00 ± 0.97	4.35 ± 0.98	13.91 ± 2.11	59.37 ± 10.04	77.55 ± 11.03	14.83 ± 3.20	0.73 ± 0.19	217.12 ± 35.39
	E2.2	1.36 ± 0.35	0.48 ± 0.10	1.75 ± 0.89	9.25 ± 1.82	0.95 ± 0.25	0.33 ± 0.03	2.81 ± 0.64	7.75 ± 1.59	7.91 ± 1.05	29.17 ± 7.92	49.98 ± 3.99	7.59 ± 1.77	0.48 ± 0.18	119.82 ± 7.66
	E2.3	1.71 ± 0.21	0.90 ± 0.10	1.07 ± 0.27	16.51 ± 4.32	11.57 ± 2.69	0.78 ± 0.18	3.90 ± 0.49	9.89 ± 2.30	19.94 ± 1.55	46.48 ± 8.75	107.96 ± 13.47	10.12 ± 0.80	0.54 ± 0.35	231.39 ± 27.28
	E2.4	1.26 ± 0.32	0.70 ± 0.16	0.56 ± 0.18	29.69 ± 1.68	13.39 ± 0.53	0.85 ± 0.09	12.10 ± 3.39	31.67 ± 4.30	34.21 ± 4.72	18.77 ± 5.34	86.28 ± 3.20	12.23 ± 3.06	1.07 ± 0.61	242.78 ± 12.51
	E3.1	1.08 ± 0.28	0.79 ± 0.05	0.49 ± 0.16	20.72 ± 2.2	10.37 ± 0.7	0.51 ± 0.08	9.45 ± 0.37	24.22 ± 1.57	20.04 ± 2.00	40.35 ± 7.84	35.03 ± 6.25	10.45 ± 1.73	0.74 ± 0.22	174.23 ± 17.36
	E3.2	3.20 ± 0.26	1.74 ± 0.26	0.43 ± 0.10	30.10 ± 6.89	14.97 ± 3.90	0.89 ± 0.03	6.22 ± 2.04	14.79 ± 3.91	17.29 ± 4.32	10.51 ± 1.06	15.61 ± 3.15	6.16 ± 1.71	0.33 ± 0.07	122.23 ± 27.65
	E3.3	4.36 ± 0.89	2.26 ± 0.54	0.74 ± 0.05	47.42 ± 9.49	25.32 ± 3.50	1.29 ± 0.21	14.28 ± 2.83	21.62 ± 3.66	26.63 ± 6.85	79.52 ± 12.98	32.67 ± 8.02	10.84 ± 2.57	0.51 ± 0.06	267.46 ± 35.72
	E3.4	10.00 ± 0.36	4.44 ± 0.86	2.88 ± 0.22	53.14 ± 8.56	29.34 ± 5.29	2.61 ± 0.20	10.97 ± 2.94	34.02 ± 8.32	39.73 ± 11.25	34.07 ± 8.17	36.04 ± 8.03	14.53 ± 2.59	0.63 ± 0.13	272.38 ± 56.22
	E4.1	3.17 ± 0.83	2.58 ± 0.64	3.36 ± 0.89	53.92 ± 4.19	28.28 ± 3.34	1.01 ± 0.05	17.25 ± 2.46	14.79 ± 1.30	30.20 ± 1.17	28.33 ± 3.81	29.47 ± 2.08	8.08 ± 1.52	1.58 ± 0.46	222.02 ± 21.34
	E4.2	2.03 ± 0.51	0.97 ± 0.22	1.03 ± 0.27	28.86 ± 4.95	16.20 ± 1.88	0.69 ± 0.11	8.10 ± 1.71	11.49 ± 2.23	16.02 ± 3.92	20.76 ± 3.10	19.29 ± 4.14	5.16 ± 1.48	0.43 ± 0.12	131.05 ± 18.89
	E4.3	4.83 ± 0.82	2.61 ± 0.54	2.11 ± 0.54	49.97 ± 12.97	25.69 ± 5.95	1.49 ± 0.23	19.11 ± 0.81	18.79 ± 3.00	37.57 ± 9.85	37.10 ± 10.3	32.84 ± 8.18	14.23 ± 2.17	1.29 ± 0.03	247.63 ± 54.41
	E4.4	1.53 ± 0.21	0.86 ± 0.18	0.53 ± 0.04	14.89 ± 2.04	9.05 ± 0.46	0.45 ± 0.05	3.29 ± 0.36	9.65 ± 0.58	11.12 ± 1.28	12.88 ± 0.77	12.46 ± 0.27	5.88 ± 0.31	0.48 ± 0.13	83.06 ± 3.83
L/S: 4/1	Control	1.35 ± 0.23	0.85 ± 0.18	0.88 ± 0.14	22.79 ± 7.12	12.16 ± 6.10	0.60 ± 0.23	10.79 ± 2.02	16.65 ± 3.86	19.83 ± 5.53	15.13 ± 4.43	20.01 ± 3.90	8.30 ± 1.78	0.72 ± 0.18	130.05 ± 22.03
	E1.1	9.80 ± 0.10	4.64 ± 0.05	2.55 ± 0.03	55.63 ± 0.56	40.49 ± 0.41	3.01 ± 0.03	11.19 ± 0.11	11.59 ± 0.12	34.96 ± 0.35	130.62 ± 1.32	65.22 ± 0.66	17.66 ± 0.18	1.31 ± 0.01	388.67 ± 3.93
	E1.2	7.34 ± 2.09	4.43 ± 0.02	1.41 ± 0.11	38.53 ± 4.44	52.49 ± 17.15	1.99 ± 0.62	12.31 ± 0.25	17.01 ± 2.86	37.21 ± 5.29	94.71 ± 16.02	26.96 ± 0.41	14.02 ± 1.36	1.30 ± 0.25	309.71 ± 15.31
	E1.3	8.37 ± 2.33	4.36 ± 0.24	1.16 ± 0.35	53.89 ± 8.77	74.64 ± 9.29	2.02 ± 0.35	10.34 ± 0.55	16.97 ± 2.26	37.60 ± 5.53	107.08 ± 31.26	25.78 ± 2.42	13.97 ± 3.64	0.59 ± 0.03	356.78 ± 42.77
	E1.4	23.97 ± 2.44	10.80 ± 0.96	9.21 ± 2.34	95.67 ± 8.69	73.73 ± 4.43	6.31 ± 0.40	27.13 ± 1.44	31.63 ± 9.13	87.61 ± 19.11	182.17 ± 8.51	46.57 ± 13.12	29.51 ± 2.10	2.77 ± 1.09	627.08 ± 71.60
	E2.1	1.47 ± 0.16	0.60 ± 0.07	1.29 ± 0.66	11.82 ± 2.74	10.04 ± 1.60	0.64 ± 0.08	4.17 ± 0.78	8.57 ± 1.46	10.41 ± 1.65	17.35 ± 4.60	13.57 ± 3.58	6.80 ± 0.79	0.14 ± 0.03	86.86 ± 8.36
	E2.2	1.72 ± 0.19	0.77 ± 0.12	1.59 ± 0.35	10.92 ± 1.10	8.06 ± 0.08	0.57 ± 0.08	3.87 ± 0.14	7.02 ± 0.36	8.21 ± 1.85	15.92 ± 3.40	12.36 ± 2.27	6.04 ± 1.72	0.24 ± 0.01	77.40 ± 10.44
	E2.3	0.20 ± 0.04	0.10 ± 0.02	1.34 ± 0.09	5.92 ± 1.16	3.90 ± 0.33	0.25 ± 0.05	3.00 ± 0.39	7.46 ± 1.68	5.76 ± 1.50	13.23 ± 2.02	14.39 ± 2.60	5.12 ± 1.24	0.36 ± 0.02	61.03 ± 7.92
	E2.4	1.54 ± 0.22	0.62 ± 0.12	1.02 ± 0.18	8.71 ± 0.98	7.25 ± 0.88	0.60 ± 0.06	3.02 ± 0.38	7.56 ± 0.89	8.21 ± 0.99	15.91 ± 2.38	13.28 ± 1.72	5.94 ± 0.69	0.19 ± 0.06	73.86 ± 8.35
	E3.1	8.16 ± 1.39	3.15 ± 0.35	0.67 ± 0.15	63.63 ± 13.36	33.83 ± 7.22	2.82 ± 0.67	8.01 ± 2.13	36.87 ± 11.02	41.24 ± 12.34	135.10 ± 40.40	44.65 ± 8.52	13.79 ± 2.85	0.53 ± 0.09	392.45 ± 47.60
	E3.2	1.48 ± 0.46	0.65 ± 0.18	0.55 ± 0.15	13.03 ± 0.31	7.99 ± 0.37	0.66 ± 0.07	4.55 ± 1.15	16.17 ± 3.41	17.61 ± 3.64	45.44 ± 11.15	18.09 ± 5.17	8.33 ± 1.98	0.16 ± 0.01	134.70 ± 24.14
	E3.3	0.85 ± 0.13	0.51 ± 0.04	0.58 ± 0.05	25.22 ± 2.66	14.63 ± 0.86	0.76 ± 0.08	12.22 ± 0.73	31.38 ± 2.66	21.35 ± 2.21	77.57 ± 13.40	53.99 ± 4.13	15.70 ± 1.30	1.15 ± 0.20	255.90 ± 27.02
	E3.4	0.77 ± 0.04	0.39 ± 0.07	0.47 ± 0.03	29.56 ± 5.54	13.67 ± 3.17	0.87 ± 0.19	10.82 ± 0.88	37.83 ± 7.67	25.67 ± 4.39	78.27 ± 22.06	43.83 ± 6.89	23.33 ± 4.24	0.33 ± 0.03	265.81 ± 55.09
	E4.1	0.45 ± 0.12	0.20 ± 0.03	1.05 ± 0.19	15.82 ± 3.04	5.75 ± 1.06	0.36 ± 0.05	6.75 ± 0.92	14.49 ± 3.27	15.26 ± 3.86	12.73 ± 2.39	20.11 ± 3.09	8.34 ± 2.12	0.32 ± 0.05	101.63 ± 19.72
	E4.2	0.85 ± 0.10	0.48 ± 0.04	0.47 ± 0.12	37.70 ± 5.14	13.80 ± 0.92	0.75 ± 0.14	20.35 ± 4.31	33.43 ± 5.01	32.90 ± 3.50	14.69 ± 0.83	38.15 ± 3.64	13.89 ± 1.08	0.99 ± 0.09	208.47 ± 20.53
	E4.3	0.10 ± 0.01	0.03 ± 0.01	2.67 ± 0.69	3.73 ± 0.44	2.44 ± 0.61	0.12 ± 0.01	2.34 ± 0.93	6.82 ± 1.52	3.06 ± 0.55	6.83 ± 0.77	8.50 ± 0.93	2.99 ± 0.78	0.85 ± 0.25	40.48 ± 3.78

	E4.4	1.42 ± 0.14	0.76 ± 0.07	0.55 ± 0.06	10.62 ± 0.97	6.91 ± 0.73	0.45 ± 0.05	6.49 ± 1.01	9.17 ± 0.83	8.88 ± 1.82	9.51 ± 1.26	13.13 ± 1.18	4.26 ± 0.37	1.12 ± 0.31	73.27 ± 7.38

[a]All parameters are given with their standard deviation (n = 3). 2-*O*-MeFuc: 2-*O*-CH_3-Fucose; 2-*O*-MeXyl: 2-*O*- CH_3-Xylose; Api: apiose; Ara: arabinose; Rha: rhamnose; Fuc: fucose; Xyl: xylose; Man: mannose; Gal: galactose; GalA: galacturonic acid; Glu: glucose; GluA: glucuronic acid; Kdo: 3-deoxyoctulosonic acid; TMS: total monosaccharides as the sum of individual monosaccharides. [b]See nomenclature in section 3.2.

Table S2. Monosaccharide composition (mg monosaccharide/g extract) of *Vitis vinifera* L. Tempranillo marc from the extractions assisted by enzymes doses E1, E2, E3, and E4 and control of *Vitis vinifera* L. Tempranillo marc at L/S ratio of 1.3/1 and 4/1.

	Sample[b]	2-*O*-MeFuc	2-*O*-MeXyl	Api	Ara	Rha	Fuc	Xyl	Man	Gal	GalA	Glu	GluA	Kdo	TMS
L/S: 1.3/1	Control	0.62 ± 0.09	0.32 ± 0.05	0.96 ± 0.74	11.60 ± 1.73	3.56 ± 0.72	.23 ± 0.05	5.44 ± 0.74	8.80 ± 2.06	5.62 ± 1.16	6.25 ± 2.71	3.56 ± 1.03	7.92 ± 2.38	0.59 ± 0.24	55.48 ± 6.8
	E1.1	2.37 ± 0.08	1.60 ± 0.03	0.64 ± 0.06	29.95 ± 0.52	10.21 ± 0.61	0.66 ± 0.01	23.06 ± 1.18	23.05 ± 0.10	19.90 ± 1.16	40.03 ± 0.75	8.39 ± 0.26	16.16 ± 0.94	1.77 ± 0.31	177.79 ± 3.35
	E1.2	13.88 ± 2.53	6.38 ± 0.95	3.98 ± 0.17	114.22 ± 16.94	43.77 ± 5.64	3.81 ± 0.56	48.91 ± 7.70	91.90 ± 7.52	71.01 ± 1.68	210.80 ± 41.45	16.82 ± 1.10	87.12 ± 15.68	1.55 ± 0.95	714.16 ± 87.49
	E1.3	4.70 ± 0.60	2.53 ± 0.17	0.89 ± 0.12	52.09 ± 5.84	17.63 ± 1.49	1.26 ± 0.27	22.40 ± 0.37	49.40 ± 9.50	30.69 ± 6.05	103.66 ± 25.09	11.61 ± 2.28	40.02 ± 11.67	0.93 ± 0.46	337.82 ± 48.63
	E1.4	13.11 ± 0.11	6.92 ± 0.94	5.22 ± 2.29	125.39 ± 8.61	45.57 ± 2.59	3.38 ± 0.11	83.20 ± 18.63	109.59 ± 15.41	77.11 ± 9.67	212.59 ± 4.95	25.13 ± 3.16	84.03 ± 9.17	1.55 ± 0.20	792.77 ± 52.92
	E2.1	5.16 ± 0.30	3.58 ± 0.52	0.89 ± 0.22	81.91 ± 8.28	27.26 ± 3.11	1.71 ± 0.21	48.44 ± 8.56	49.22 ± 5.37	53.49 ± 4.51	82.83 ± 27.43	27.04 ± 2.17	23.99 ± 9.71	1.67 ± 0.74	407.19 ± 34.16
	E2.2	1.53 ± 0.32	1.00 ± 0.26	0.48 ± 0.21	24.28 ± 5.29	7.68 ± 1.58	0.47 ± 0.08	13.35 ± 3.75	15.58 ± 3.55	17.54 ± 5.04	26.57 ± 9.08	13.46 ± 2.68	10.04 ± 4.28	1.16 ± 0.24	133.14 ± 28.81
	E2.3	6.18 ± 0.89	2.97 ± 0.32	1.44 ± 0.43	85.48 ± 7.36	28.2 ± 1.91	1.90 ± 0.15	33.23 ± 5.43	74.91 ± 4.03	55.75 ± 3.05	40.93 ± 7.08	16.57 ± 2.01	62.94 ± 3.92	0.56 ± 0.18	411.06 ± 23.32
	E2.4	2.12 ± 0.24	0.92 ± 0.01	0.85 ± 0.44	36.96 ± 0.46	9.79 ± 0.58	0.70 ± 0.03	9.51 ± 2.22	19.24 ± 1.51	15.99 ± 4.40	45.34 ± 5.38	9.47 ± 2.19	14.02 ± 2.62	0.96 ± 0.17	165.85 ± 5.96
	E3.1	0.84 ± 0.14	0.40 ± 0.07	0.48 ± 0.12	24.86 ± 7.07	7.61 ± 1.41	0.42 ± 0.09	12.50 ± 2.38	24.10 ± 4.03	17.07 ± 3.26	51.00 ± 5.51	3.33 ± 0.70	27.56 ± 3.28	0.14 ± 0.06	170.31 ± 26.43
	E3.2	1.55 ± 0.40	0.79 ± 0.23	0.34 ± 0.11	19.95 ± 4.68	7.94 ± 1.23	0.50 ± 0.12	10.60 ± 0.93	13.13 ± 2.93	11.34 ± 2.23	36.16 ± 7.31	4.59 ± 0.58	10.60 ± 0.42	0.65 ± 0.06	118.13 ± 16.25
	E3.3	0.98 ± 0.10	0.60 ± 0.08	0.44 ± 0.06	13.79 ± 2.63	5.97 ± 0.57	0.35 ± 0.05	9.03 ± 0.50	13.13 ± 3.48	9.80 ± 3.04	8.01 ± 1.72	4.99 ± 0.31	14.82 ± 2.70	0.19 ± 0.05	82.13 ± 4.69
	E3.4	2.51 ± 0.14	1.07 ± 0.23	0.27 ± 0.02	27.93 ± 6.53	9.29 ± 0.93	0.73 ± 0.06	8.96 ± 0.77	22.56 ± 7.74	15.29 ± 5.15	57.84 ± 5.18	7.46 ± 2.75	18.24 ± 3.88	0.34 ± 0.03	172.50± 30.87
	E4.1	1.66 ± 0.45	0.81 ± 0.19	0.40 ± 0.07	27.59 ± 7.79	9.10 ± 1.73	0.70 ± 0.13	13.10 ± 1.04	35.75 ± 8.26	26.74 ± 5.76	75.27 ± 8.99	9.47 ± 2.12	35.12 ± 6.48	0.26 ± 0.04	235.98 ± 19.90
	E4.2	0.46 ± 0.01	0.24 ± 0.03	1.29 ± 0.33	10.59 ± 0.51	2.96 ± 0.40	0.18 ± 0.02	6.98 ± 1.79	10.21 ± 1.09	6.70 ± 0.36	16.66 ± 3.08	3.35 ± 0.37	6.20 ± 1.12	0.34 ± 0.05	66.15 ± 2.54
	E4.3	1.68 ± 0.43	0.97 ± 0.24	1.27 ± 0.21	40.75 ± 8.50	9.59 ± 1.14	0.57 ± 0.11	16.51 ± 2.62	40.60 ± 8.92	25.17 ± 3.12	59.80 ± 7.30	8.32 ± 1.55	35.45 ± 3.77	0.47 ± 0.04	241.14 ± 34.76
	E4.4	0.46 ± 0.08	0.22 ± 0.03	0.96 ± 0.69	9.92 ± 1.49	2.74 ± 0.39	0.16 ± 0.03	5.49 ± 0.70	8.09 ± 0.57	4.00 ± 0.96	19.23 ± 4.65	3.56 ± 0.81	8.24 ± 0.53	0.25 ± 0.05	63.34 ± 7.05
L/S: 4/1	Control	0.31 ± 0.14	0.14 ± 0.08	0.98 ± 0.29	8.09 ± 3.34	2.42 ± 1.15	0.17 ± 0.09	4.73 ± 2.17	7.94 ± 3.28	4.52 ± 2.69	6.06 ± 3.08	2.06 ± 1.23	5.87 ± 0.55	0.99 ± 0.62	44.26 ± 13.91
	E1.1	8.46 ± 1.19	4.88 ± 0.73	1.86 ± 1.03	68.53 ± 9.77	33.52 ± 5.30	2.44 ± 0.32	58.59 ± 16.97	65.10 ± 5.87	42.81 ± 6.55	152.63 ± 45.60	8.62 ± 1.33	23.77 ± 5.62	1.55 ± 0.67	472.76 ± 60.16
	E1.2	13.13 ± 3.42	6.11 ± 1.25	3.04 ± 0.46	77.90 ± 7.89	43.40 ± 8.75	3.47 ± 0.38	54.18 ± 10.35	62.27 ± 8.13	38.14 ± 3.57	158.92 ± 1.03	7.61 ± 0.87	55.64 ± 6.62	0.78 ± 0.18	524.59 ± 26.07
	E1.3	12.57 ± 1.51	5.62 ± 0.45	2.93 ± 2.01	75.68 ± 6.88	39.80 ± 5.00	3.54 ± 0.41	52.73 ± 3.14	77.47 ± 12.68	40.78 ± 7.67	201.99 ± 45.61	8.62± 1.57	47.65 ± 23.89	1.47 ± 0.46	570.84 ± 96.99
	E1.4	7.06 ± 0.60	3.29 ± 0.23	1.97 ± 0.82	34.88 ± 0.97	21.62 ± 1.59	1.73 ± 0.14	26.83 ± 0.56	23.57 ± 2.35	14.28 ± 1.20	84.89 ± 11.45	4.70 ± 0.73	26.02 ± 1.71	1.45 ± 0.45	252.29 ± 10.59
	E2.1	1.05 ± 0.21	0.63 ± 0.11	0.48 ± 0.08	22.00 ± 5.83	5.96 ± 1.67	0.39 ± 0.09	10.97 ± 2.52	13.07 ± 1.96	11.36 ± 1.42	15.26 ± 1.40	3.69 ± 0.89	7.78 ± 1.18	1.03 ± 0.18	93.67 ± 13.97
	E2.2	0.41 ± 0.03	0.24 ± 0.04	0.26 ± 0.02	23.48 ± 4.63	4.04 ± 0.61	0.27 ± 0.02	10.97 ± 4.05	14.21 ± 1.61	9.46 ± 0.59	17.10 ± 8.02	1.86 ± 0.26	8.23 ± 4.90	0.69 ± 0.45	91.22 ± 4.95
	E2.3	1.08 ± 0.17	0.68 ± 0.06	0.37 ± 0.06	16.36 ± 5.16	5.41 ± 0.44	0.34 ± 0.03	11.00 ± 0.94	9.79 ± 0.81	9.20 ± 0.82	12.30 ± 2.61	2.10 ± 0.18	3.91 ± 1.64	1.56 ± 0.22	74.09 ± 7.75
	E2.4	1.01 ± 0.05	0.53 ± 0.04	0.25 ± 0.02	8.68 ± 0.40	4.50 ± 0.43	0.31 ± 0.02	6.43 ± 1.59	10.20 ± 1.08	6.10 ± 0.46	7.88 ± 0.36	1.56 ± 0.20	7.38 ± 1.11	0.19 ± 0.04	55.02 ± 5.13
	E3.1	1.83 ± 0.28	0.77 ± 0.03	0.38 ± 0.12	32.27 ± 3.58	10.37 ± 1.95	0.74 ± 0.10	12.74 ± 0.34	27.19 ± 1.81	14.32 ± 0.35	56.99 ± 2.36	3.32 ± 0.80	23.75 ± 0.39	0.40 ± 0.17	185.07 ± 6.33
	E3.2	0.15 ± 0.04	0.09 ± 0.02	0.95 ± 0.21	6.45 ± 0.10	1.73 ± 0.02	0.13 ± 0.00	4.82 ± 0.26	7.95 ± 0.27	3.51 ± 0.02	3.49 ± 0.01	2.27 ± 0.17	5.58 ± 0.81	0.13 ± 0.03	37.25 ± 0.32
	E3.3	0.23 ± 0.07	0.05 ± 0.00	0.84 ± 0.19	7.19 ± 0.95	1.96 ± 0.27	0.17 ± 0.03	3.71 ± 0.16	9.09 ± 2.38	4.10 ± 0.99	5.02 ± 1.25	2.54 ± 0.32	7.12 ± 1.56	0.16 ± 0.03	42.20 ± 7.78
	E3.4	0.07 ± 0.02	0.03 ± 0.00	0.13 ± 0.01	2.94 ± 0.37	1.51 ± 0.04	0.08 ± 0.01	2.54 ± 0.30	4.31 ± 0.50	1.16 ± 0.02	8.20 ± 0.09	1.21 ± 0.09	3.32 ± 0.72	0.04 ± 0.01	25.54 ± 0.62
	E4.1	0.23 ± 0.04	0.13 ± 0.03	0.19 ± 0.02	4.02 ± 0.67	0.99 ± 0.27	0.07 ± 0.01	1.86 ± 0.41	3.40 ± 0.29	1.93 ± 0.49	1.57 ± 0.10	0.98 ± 0.20	2.06 ± 0.02	0.41 ± 0.11	17.84 ± 2.42
	E4.2	0.04 ± 0.01	0.03 ± 0.01	0.80 ± 0.13	2.90 ± 0.35	0.66 ± 0.17	0.06 ± 0.01	1.69 ± 0.19	3.02 ± 0.86	0.99 ± 0.24	4.98 ± 0.44	0.81 ± 0.13	1.95 ± 0.13	0.12 ± 0.02	18.04 ± 2.30
	E4.3	0.10 ± 0.02	0.06 ± 0.02	1.03 ± 0.28	6.11 ± 0.98	1.44 ± 0.29	0.11 ± 0.02	4.28 ± 0.61	8.16 ± 0.93	2.65 ± 0.29	17.9 ± 1.95	3.05 ± 0.97	7.93 ± 0.88	0.15 ± 0.03	52.97 ± 6.64
	E4.4	0.19 ± 0.02	0.11 ± 0.01	1.92 ± 0.17	15.42 ± 1.94	1.53 ± 0.17	0.10 ± 0.00	6.68 ± 1.93	6.66 ± 0.98	1.76 ± 0.25	9.98 ± 2.45	3.05 ± 0.55	2.89 ± 0.36	0.90 ± 0.13	51.20 2.82

[a]All parameters are given with their standard deviation (n = 3). 2-*O*-MeFuc: 2-*O*-CH_3-Fucose; 2-*O*-MeXyl: 2-*O*- CH_3-Xylose; Api: apiose; Ara: arabinose; Rha: rhamnose; Fuc: fucose; Xyl: xylose; Man: mannose; Gal: galactose; GalA: galacturonic acid; Glu: glucose; GluA: glucuronic acid; Kdo: 3-deoxyoctulosonic acid; TMS: total monosaccharides as the sum of individual monosaccharides. [b]See nomenclature in section 3.2.

Figure S1. HPSEC-RID chromatograms of total soluble polysaccharides in the extracts from enzyme-assisted extraction (EAE) in *Vitis vinifera* L. Viura pomace compared to the control sample at liquid/solid ratios (L/S) of 1.3/1 and 4/1. Chromatograms obtained using two serial Shodex OHpack KB-803 and KB-805 columns. HMW: high molecular weight fraction (20–400 kDa); MMW: medium molecular weight fraction (5.9–20 kDa); LMW: low molecular weight fraction (<5.9 kDa); E1, E2, E3, and E4: See nomenclature in Section 3.2.

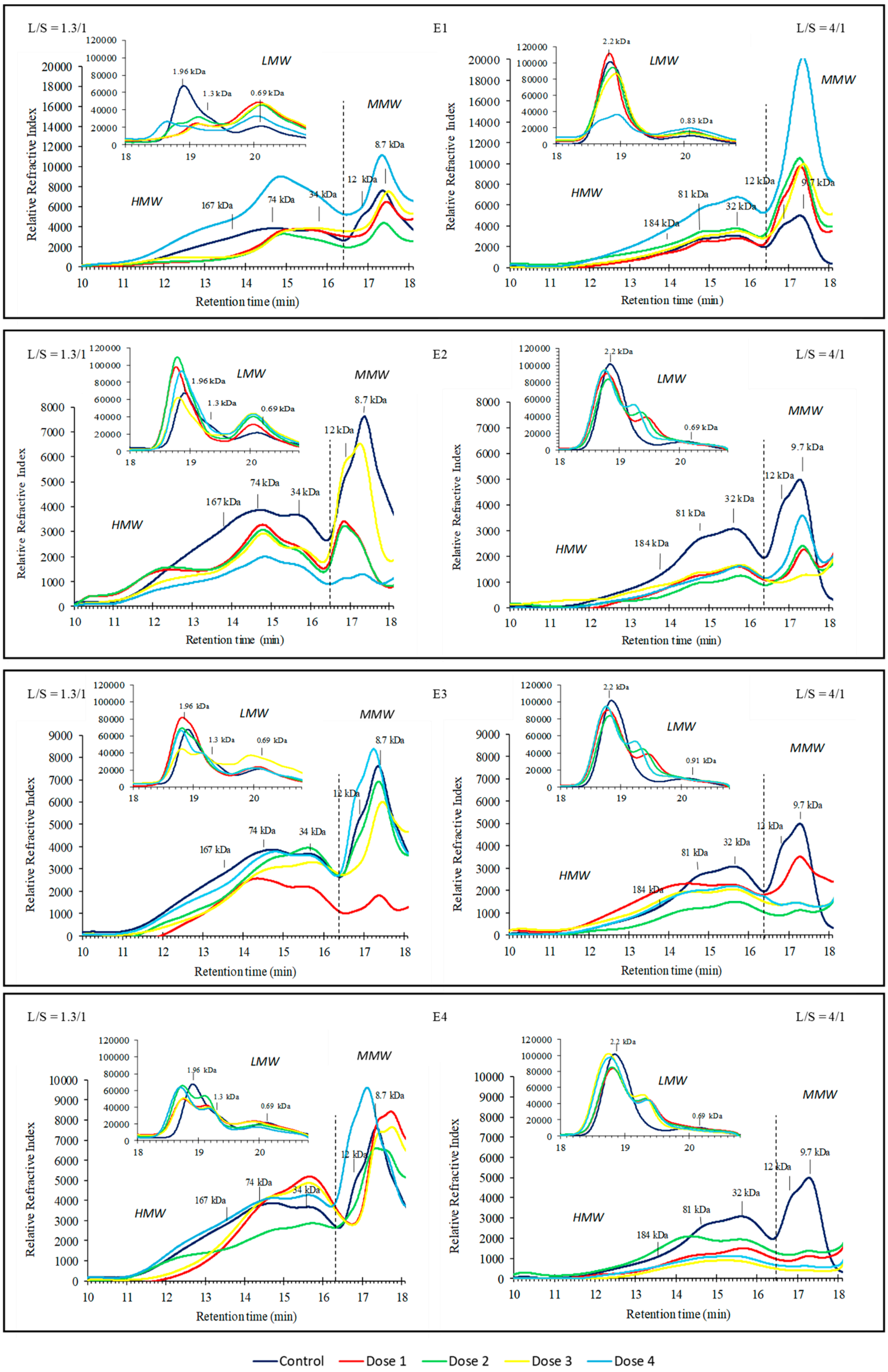

Figure S2. HPSEC-RID chromatograms of total soluble polysaccharides in the extracts from enzyme-assisted extraction (EAE) in *Vitis vinifera* L. Tempranillo marc compared to the control samples at liquid/solid ratio (L/S) of 1.3/1 and 4/1. Chromatograms obtained using two serial Shodex OHpack KB-803 and KB-805 columns. HMW: high molecular weight fraction (20–400 kDa); MMW: medium molecular weight fraction (5.9–20 kDa); LMW: low molecular weight fraction (<5.9 kDa); E1, E2, E3, and E4: See nomenclature in Section 3.2.

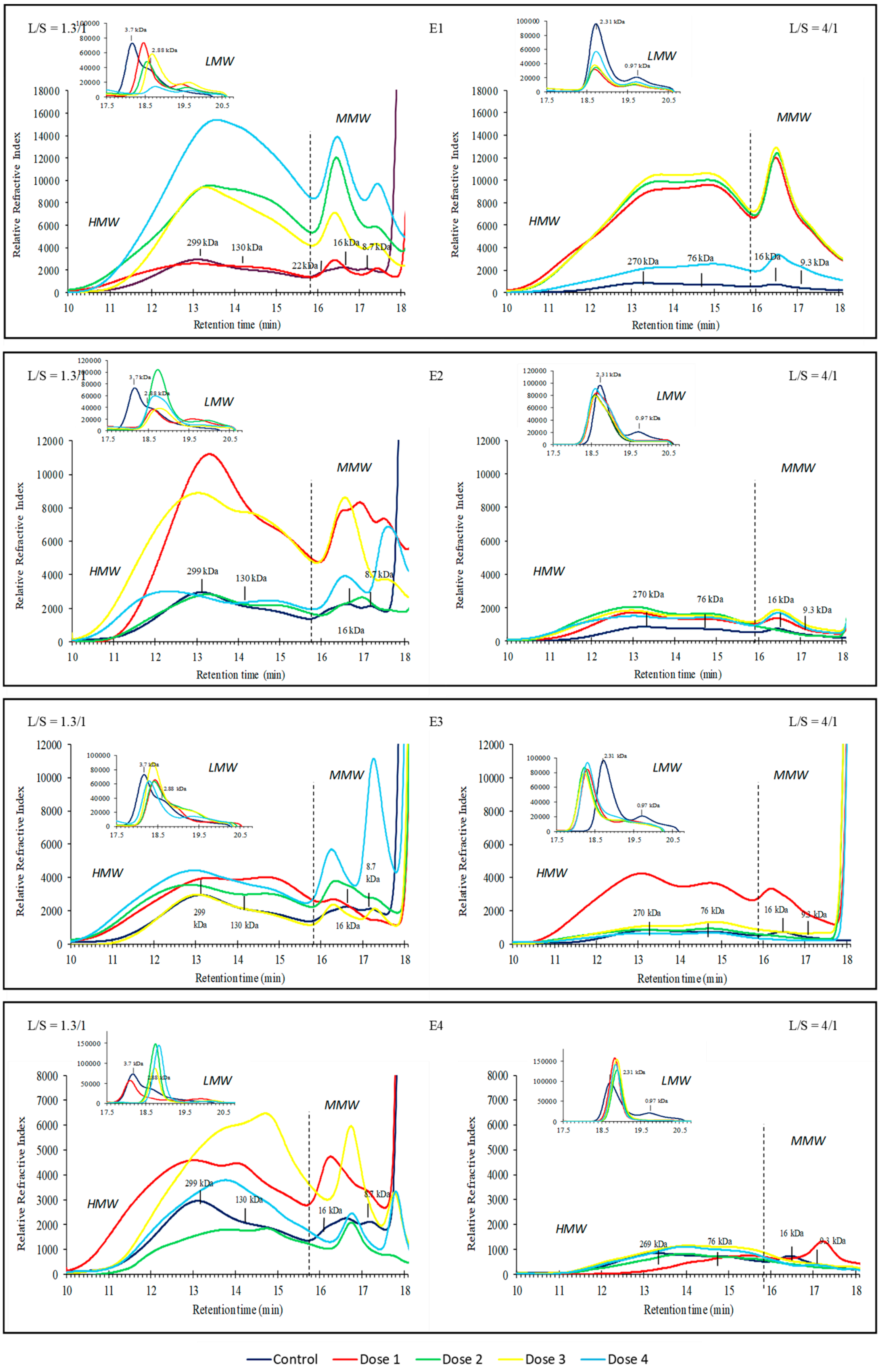

Supplement: Supplementary file 1 [file molecules-30-00213-s001.zip › molecules-3367569-supplementary.pdf]
